# Optimization of indirect wastewater characterization using led spectrophotometry: a comparative analysis of regression, scaling, and dimensionality reduction methods

**DOI:** 10.1007/s11356-024-34714-8

**Published:** 2024-08-28

**Authors:** Daniel Carreres-Prieto, Enrique Fernandez-Blanco, Daniel Rivero, Juan R. Rabuñal, Jose Anta, Juan T. García

**Affiliations:** 1 Department of Engineering and Applied Techniques, Centro Universitario de la Defensa, Universidad Politécnica de Cartagena, C/ Coronel López Peña S/N, Base Aérea de San Javier, Santiago de La Ribera, 30720 Murcia, Spain; 2 Department of Computer Science and Information Technologies, Universidade da Coruña, CITIC, 15071 A Coruña, Spain; 3 Artificial Neural Networks and Adaptative Systems Research Group (RNASA) and Centre of Technological Innovation in Construction and Civil Engineering (CITEEC), University of A Coruña, 15071 A Coruña, Spain; 4 Water and Environmental Engineering Research Team (GEAMA), Civil Engineering School, Universidade da Coruña, CITEEC, 15071 A Coruña, Spain; 5 Department of Mining and Civil Engineering, Universidad Politécnica de Cartagena, 30202 Cartagena, Spain

**Keywords:** Wastewater characterization, LED spectrophotometer, Wastewater quality, Comparison between characterization techniques, Chemical Oxygen of Demand, Total suspended solids

## Abstract

LED spectrophotometry is a robust technique for the indirect characterization of wastewater pollutant load through correlation modeling. To tackle this issue, a dataset with 1300 samples was collected, from both raw and treated wastewater from 45 wastewater treatment plants in Spain and Chile collected over 4 years. The type of regressor, scaling, and dimensionality reduction technique and nature of the data play crucial roles in the performance of the processing pipeline. Eighty-four pipelines were tested through exhaustive experimentation resulting from the combination of 7 regression techniques, 3 scaling methods, and 4 possible dimensional reductions. Those combinations were tested on the prediction of chemical oxygen demand (COD) and total suspended solids (TSS). Each pipeline underwent a tenfold cross-validation on 15 sub-datasets derived from the original dataset, accounting for variations in plants and wastewater types. The results point to the normalization of the data followed by a conversion through the PCA to finally apply a Random Forest Regressor as the combination which stood out These results highlight the importance of modeling strategies in wastewater management using techniques such as LED spectrophotometry.

**Supplementary Information:**

The online version contains supplementary material available at 10.1007/s11356-024-34714-8.

## Introduction

Effective water quality monitoring is essential for resource management and environmental protection (Sun et al. 2023 and Shao et al. 2021). Over the past decade, molecular spectrophotometry has emerged as a dependable method for indirectly characterizing pollutants in wastewater. Through this technique, real-time and online monitoring of the entire wastewater network has become feasible. Numerous contributions have provided proof on this topic (Altmann et al. 2016; Lepot et al. 2016; Mesquita et al. 2017; Korshin et al. 2018, Brito et al. 2014, Feng et al. 2018, and Cheng et al. 2022 or Song et al. 2021), reporting the effectiveness of molecular spectrophotometry in identifying and quantifying pollutants in wastewater.

The physicochemical properties of wastewater significantly influence its spectral response, allowing the detection of parameters such as chemical oxygen demand (COD), total suspended solids (TSS), and total nitrogen (TN), among others (Ferree and Shannon 2001; Rieger et al. 2004; Korshin et al. 2018; and Carreres-Prieto et al. 2023a). This is because the chemical and molecular composition of the wastewater directly affects the interaction of light with the sample.

Light absorption and scattering vary according to the dissolved and suspended compounds in the water (Lourenço, et al. 2010); their organic, inorganic, and biological characteristics determine specific absorption and scattering patterns. In addition, interactions within the aqueous matrix, such as the formation of molecular complexes or changes in the molecular structure due to the presence of various ions and materials, modify the optical behavior of contaminants. Interactions between different dissolved molecules can change the optical properties of the solution, while oxidation or reduction processes alter the oxidation state of certain contaminants, which affects their light absorption. These factors create a unique spectral profile for each wastewater sample, allowing the development of accurate and robust predictive models for efficient wastewater management.

This indirect characterization is carried out using correlation models, where research works such as Lepot et al. (2016), Torres (2008), Piro et al. (2012), Qin et al. (2012), or Carreres-Prieto et al. (2023b) have verified the adequacy of these characterization models.

However, the performance of a model is determined by several factors, such as the preprocessing and/or scaling applied to the data or the regression technique employed. Additionally, the nature of the spectral data itself can have a major impact depending on the wastewater matrix or the geographical area where they have been taken from population, presence and type of industrial activity, and purification conditions, among others.

Therefore, this work proposes an in-depth study of this issue considering two objective pollutants, COD and TSS, registered in about 1300 wastewater samples from 45 WWTPs collected over 4 years. The gathered data corresponding with the spectrum recorded from the visible field (380–700 nm) was used to determine which pipeline composed of regressor, scaling, and dimensionality reduction techniques is the most appropriate to tackle the characterization of the pollutant load in the wastewater. This characterization was carried out regardless of the type of water to be studied (raw wastewater, treated wastewater, or a combination of both), as well as the area or WWTP to which it belongs and for each particular case of WWTP and type of water.

In this study, not only did we seek to demonstrate the suitability of LED spectrophotometry as an indirect water analysis technique, but a comprehensive approach was adopted by testing a wide range of preprocessing and dimensionality reduction techniques, along with various regression algorithms. The objective was to evaluate all possible combinations of these methodologies in different scenarios to determine which one optimized performance by minimizing the root mean square error (RMSE). In order that the evaluation was agnostic of the data used for the test, it was made following a cross-validation schema. This evaluation has been carried out for both the overall set of all water and treatment plants as well as individually for each one.

LED spectrophotometry has proven to be a suitable analytical technique to carry out studies on the properties of both drinking water, as demonstrated by works such as Bridgeman et al. (2015) or Prairie et al. 2020, and wastewater (Place 2019 or Carreres-Prieto et al. 2023a, 2023b). The main novelty of the present work lies in the exhaustive development and evaluation of a spectral data analysis pipeline including modeling, scaling, and dimension reduction steps for more than 1300 sample datasets with different wastewater matrices.

The relevance of this study is based on its ability to significantly improve wastewater management in terms of COD and TSS levels. By identifying the most effective combinations of scaling, dimensionality reduction, and regression techniques, this work will provide a solid foundation for accurate spectral analysis of wastewater. This is crucial as accurate pollutant characterization is critical for the design and optimization of more effective and sustainable water treatment strategies (Orhon et al. 1997; Rosal et al. 2010; Saravanan et al. 2021; Muralikrishna and Manickam 2017). Moreover, the findings of this research will have a direct impact on environmental protection and public health by enabling more accurate and reliable water quality monitoring.

The current study presents an advanced methodology in wastewater treatment modeling, based on the analysis of an extensive collection of data collected from a large number of WWTPs from different locations, with wastewater samples of differing compositions, obtained over several years, thus reflecting the temporal variation of the pollutant load and its diversity depending on the type of water and location it originates from. Additionally, a large number of techniques and combinations were used to carry out the study. The inclusion of a wide range of data, including statistical outliers, has enabled the development of high-performance predictive models, characterized by their robustness and applicability under various conditions. This innovative approach overcomes the limitations of previous studies, offering generalizable models with low error levels, which can cope with real operating conditions, thereby representing a significant step towards the optimization of WWTPs.

The rest of the manuscript is organized as follows:

“Materials and methods” section details the experimental campaign carried out, describes the regression, scaling, and dimensionality reduction techniques, and presents an outline of the work carried out.

“Results and discussion” section shows, for both COD and TSS, the analyses carried out for each of the water types and WWTPs, both individually and jointly, to determine, respectively, which type of regressor, scaling, and dimensionality reduction technique is the most appropriate to apply to achieve the best models for characterizing the pollutant load of wastewater, regardless of the type of wastewater and WWTP that they come from.

Finally, “Conclusions” section outlines the main conclusions reached in this research work.

## Materials and methods

### Experimental campaign

**Fig. 1:**
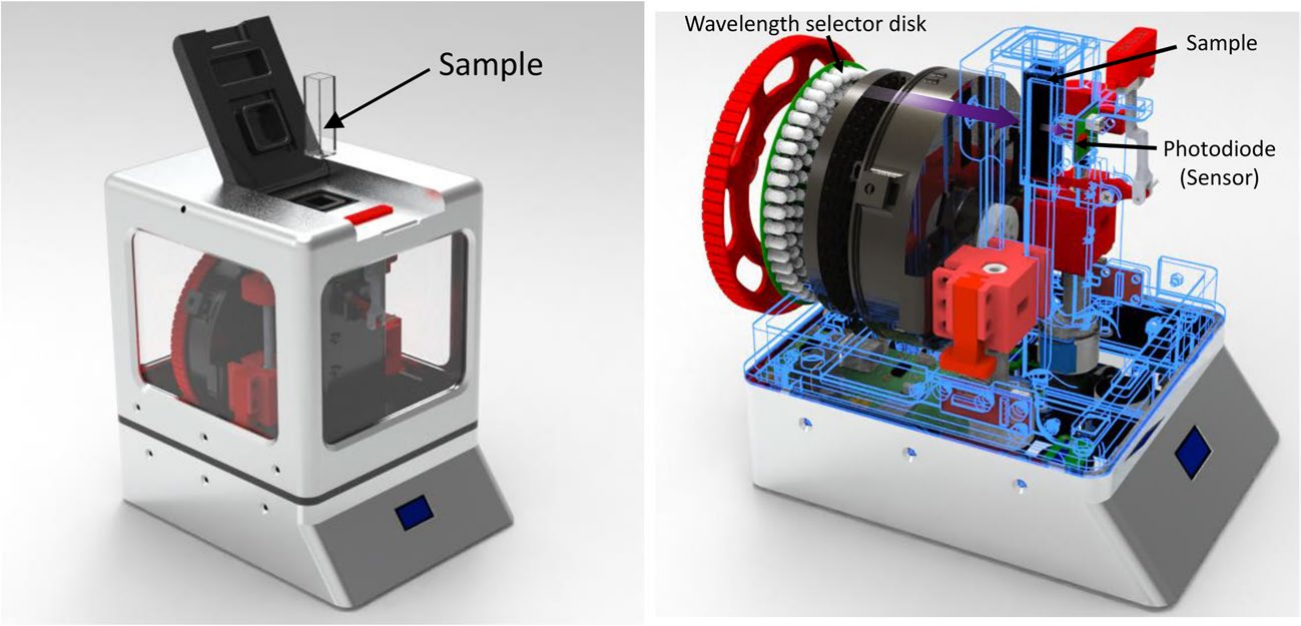
LED spectrophotometry equipment used (Carreres-Prieto et al. 2019)

For the present study, 1300 wastewater samples, both raw and after a secondary treatment, were collected over 4 years from 45 WWTPs located in northwestern and southeastern Spain and in Chile, to contribute to the treatment objectives defined in directive 91/271 EEC concerning urban wastewater treatment. These samples represent a 24-h integration and were analyzed using LED spectrophotometry equipment (Carreres-Prieto et al. 2019) operating in the 380–700 nm range (Fig. 1), in order to obtain the spectral response, without employing filtering or chemical reagents, and additionally the analysis of laboratory water properties, to obtain COD and TSS levels and then generate correlation models for indirect characterization. The choice of COD and TSS as key variables in this study is based on their ability to provide a comprehensive assessment of the pollutant load in wastewater. COD is a critical indicator of the amount of organic matter present, providing a direct measure of the potential pollutant that can adversely affect water quality and aquatic life. On the other hand, TSS is essential for determining water clarity and the presence of suspended particles that can carry pathogens, heavy metals, and nutrients, exacerbating eutrophication problems and affecting treatment processes.

This research work used, on the one hand, the results of the spectral analysis of the samples using LED technology in the 380–700 nm range, which produced a total of 81 transmittance values and 81 absorbance values per sample, and on the other hand, the analytical values measured in the laboratory in terms of COD and TSS concentration of the water, so as to generate models that, from the spectral response, can determine the concentration of these pollutants in a simple, fast, and accurate way without the need to alter the samples or use chemical reagents. To carry out this study, a low-cost spectrophotometry equipment developed by the authors of this manuscript (Carreres-Prieto et al. 2019), which operates with LED technology, was used. This equipment, shown in Fig. 1A, can perform multispectral analysis in the mentioned range, with an accuracy comparable to that of commercial equipment based on incandescent lamps.

The LED spectrophotometer, illustrated in Fig. 1B, consists of a disc where 5 mm diameter LEDs are aligned with the sample to be analyzed. The sample is inserted through the top using a standard spectrophotometric test tube with a volume of 2.7 ml. This configuration has made it possible to achieve a working range between 380 and 700 nm using only 33 LEDs covering different areas of the visible spectrum. The choice of LED technology for this spectrophotometer is based on several significant advantages over conventional equipment based on UV–vis lamps. These advantages include low energy consumption, zero heat generation, low cost, and the ability to be used immediately, since, unlike incandescent lamps, they reach their nominal speed almost instantaneously, which translates into shorter analysis times and no heating problems, to which must be added the possibility of miniaturization, which makes LED technology an attractive option for the development of more efficient and versatile spectrophotometry equipment.

The samples were organized into four datasets, each one grouped together samples related to a specific geographic area and/or set of specific WWTPs; Table 7 of Appendix shows information on the different WWTPs that comprise it, as well as their main characteristics. Likewise, to carry out a study with a greater variety of samples from different WWTPs, a dataset called “All Datasets” was generated, which brought the four previous datasets together. In turn, each of the datasets was divided, according to the type of wastewater, into only samples of raw wastewater (Influent); only samples of treated wastewater (Effluent); and a combination of both (Both), in order to analyze the performance of the regressors, scaling and dimensionality reduction technique for each of the types of water, giving rise to a total of 15 sub-datasets.

Tables 1 and 2 show the different datasets used in this research work to generate the different correlation models for COD and TSS, respectively, as well as the total number of samples and their segregation into raw wastewater (influent) and treated water (effluent), sub-datasets, and mean, maximum, and minimum values recorded in each dataset, as well as the standard deviation of the data.

**Table 1:** Characteristics of the different datasets used for the different chemical oxygen demand (COD) estimation models

Dataset	No. of WWTPs	Sub*	Type of wastewater	No. of samples	Mean (mg/L)	Std. dev. (mg/L)	Max value	Min value
Dataset A	1	A1	Both	174	512.74	511	1672	27
		A2	Influent	86	988.62	279.84	1672	44
		A3	Effluent	88	47.67	10.27	78	27
Dataset B	42	B1	Both	420	335.64	400.25	1574	0
		B2	Influent	206	660.94	344.06	1574	14
		B3	Effluent	214	22.51	18.79	200	0
Dataset C	1	C1	Both	332	257.13	277.84	921	0
		C2	Influent	116	607.76	175.02	921	0
		C3	Effluent	216	68.82	22.55	155	5
Dataset D	1	D1	Both	346	360.28	342.68	1226	18
		D2	Influent	173	666.93	214.9	1226	40
		D3	Effluent	173	53.63	14.14	92	18
All Datasets	45	L1	Both	1272	346.08	382.48	1672	0
		L2	Influent	581	700.61	296.66	1672	0
		L3	Effluent	691	47.98	26	200	0

*Sub-datasets

**Table 2:** Characteristics of the different datasets used for the different total suspended solid (TSS) estimation models

Dataset	No. of WWTPs	Sub*	Type of wastewater	No. of samples	Mean (mg/l)	Std. dev. (mg/l)	Max value	Min value
Dataset A	1	A1	Both	156	179.45	200.85	556	3
		A2	Influent	68	392.49	107.63	556	25
		A3	Effluent	88	14.83	8.04	51	3
Dataset B	42	B1	Both	406	116.11	140.79	550	0
		B2	Influent	192	238.35	115.87	550	12
		B3	Effluent	214	6.44	9.8	90	0
Dataset C	1	C1	Both	285	86.69	113.43	356	10
		C2	Influent	69	272.42	82.09	356	17
		C3	Effluent	216	27.37	16.05	132	10
Dataset D	1	D1	Both	345	166.7	182.83	800	0
		D2	Influent	172	321.84	137.55	800	0
		D3	Effluent	173	12.46	6.84	37	0
All datasets	45	L1	Both	1192	132.01	161.04	800	0
		L2	Influent	501	292.63	130.02	800	0
		L3	Effluent	691	15.55	14.2	132	0

*Sub-datasets

Samples were taken at the inlet (pre-treatment) and outlet of the WWTPs to analyze different levels of contamination. Integrated samples were used over 24 h and collected automatically by the WWTPs’ equipment.

For this study, integrated sampling was carried out over 24 h in each of the defined measurement zones at the wastewater treatment plants (WWTPs). Each sample was analyzed using two different approaches: the developed LED spectrophotometry equipment and standard laboratory analysis methods. This allowed the results obtained by both methods to be correlated. The samples analyzed with the LED spectrophotometry kits were not subjected to pre-treatment or addition of chemical reagents, to develop an operational system with raw samples, suitable for continuous monitoring.

The laboratory tests were carried out according to the Standard Methods (SM) and the norms of the International Organisation for Standardisation (ISO), using mainly the following procedures: the dichromate method with UV–VIS spectroscopy (ISO 6060:1989) for COD and the settleable solids method (ME 2540 F) in the case of TSS.

### Model performance indicators

Model assessment was performed in terms of the RMSE (Eq. (3)), where, to analyze the performance of regressors, the dimensionality reduction technique and scaling were used separately for each sub-dataset; the median value was taken to avoid possible deviations of the results as a consequence of the random rearrangement of the data.

The *R*^2^ is a widely used performance indicator, but it has certain limitations, such as the fact that it does not take into account the scale of the data, which means that comparisons between models of different sub-datasets may be invalid. Moreover, in the case of working with new data never seen before by the models (third-party data), in the case of non-linear data or complex relationships, it may underestimate the performance of the model, since it is designed under the assumption of linearity. For this reason, RMSE was used as the main performance indicator. However, the results in terms of *R*^2^ (Eq. (4)) are also presented in the Supplementary Information to provide a broader view of the effectiveness of the models.

3 $$RMSE\;(mg/L)=\sqrt{\frac{1}{n}{\sum }_{i}^{n}{\left({X}_{G{T}_{i}}-{X}_{estimate{d}_{i}}\right)}^{2}}$$

4 $$\overline{{R }^{2}}(\%)=1-\frac{\frac{\sum_{i}^{n}{\left({X}_{G{T}_{i}}-{X}_{estimate{d}_{i}}\right)}^{2}}{n}}{\frac{\sum_{i}^{n}{\left({X}_{G{T}_{i}}-\overline{{X }_{G{T}_{i}}}\right)}^{2}}{n}}*100$$

where *n* represents the count of the samples, while ${X}_{GT}$ and ${X}_{estimated}$ are the respective measurements of the polluting parameters obtained by the analytical methods used by the wastewater treatment plant (ground truth) and by the calculation models, respectively, and $\overline{{X }_{GT}}$ is the average of the reference values.

### Methodology for data analysis

The ability to provide an accurate characterization relies on two elements, the processing pipeline model and the data used on that pipeline to assess its performance. This means that it is not enough merely to determine the different steps of the processing pipeline model, but also which data was presented to that combination to fully understand the results. In this work, the processing pipeline was set with three steps, named scaling, dimensional reduction, and regressor. A set of different techniques has been applied for each one of them; these are detailed below:

1. Scaling

- *No scaling*: the data is not transformed, and the original data is used to determine the influence of the transformation on the performance of the different models.
- *Normalization*: it brings the data to the interval [0,1] by mean (Eq. (1)) for each feature, where represents the transformed feature $(x)$, represents the minimum value, $min\left(x\right)$ among all samples and the maximum, $\text{max}\left(x\right)$.
  1 $${x}_{t} =\frac{x-min\left(x\right)}{\text{max}\left(x\right)-\text{min}\left(x\right)}$$
- *Standardization*: data is transformed to a normal distribution [− 1, 1], according to Eq. (2), where ${x}_{t}$ represents the transformed feature, ${\mu }_{x}$ represents the mean, and ${\sigma }_{x}$ the standard deviation of the whole dataset
  2 $${x}_{t}=\frac{x-{\mu }_{x}}{{\sigma }_{x}}x$$

2. Dimensional reduction

- *No transformation*: the data is not transformed from the output of the scaling step. This is to determine the influence of any possible transformation and reduction in the performance of the final step, the regressor.
- *Principal components analysis* (*PCA*): the aim of this reduction dimensionality is to transform the original variables into a new set of uncorrelated variables. Those new variables, obtained through linear combinations of the original variables, are arranged in order of their ability to account for variance in the data (Abdi and Williams 2010).
- *F-score*: it is a linear regression with feature selection that more specifically uses the Lasso or L1 method. It tries to identify the most relevant features for the target variable based on the premise that input variables with a higher correlation with the target variable have a higher significance for the model. This is determined by a cross-correlation analysis between a predictor variable and the target variable, using the *p*-value statistic to assess significance (Zhao et al. 2017, Zou 2006).
- *The Mutual Information* (*MI*) measure is used to assess the dependency between variables, and it allows for the removal of those that exceed a predetermined threshold (Seth and Príncipe 2010).

Details of the setting parameters of the dimensional reduction processes are shown in Table 3.

3. Regressors

- Partial least square (PLS): A regression technique that models linear relations between input and output variables, seeking the most appropriate subset of variables for the model based on their importance (Geladi and Kowalski 1986).
- Stochastic gradient descent (SGD): An optimization algorithm that updates model weights iteratively using small data sets to train regression models (Bottou. 2012).
- Support vector regression (SVR): Based on the behavior of support vector machines, it is a machine learning algorithm regression task. For regression, they seek to find an optimal hyperplane that approximates the relationship between input variables and their corresponding target values.
- Regression ensemble methods:

- Random Forest (RF): Classical ensemble regression technique that leverages decision trees for prediction. In this approach, each tree is trained on the entire dataset without partitions, deviating from some other methods. The final prediction is obtained by aggregating the outputs of the constituent trees within the forest. This methodology yields robust and accurate regression results (Breiman. 2001).
- Bagging Tree Regressor: Also a Decision Tree-based ensemble approach which *follows* the principle of the knowledge of the crowd. It uses a Bootstrapping or Bagging approach to build random partitions to train each of the regressors which conform the ensemble. *The results would be the average of the partial regressors* (Breiman 1996).
- Gradient Boosting (Boosting): This ensemble approach uses the 11 decision trees as the base model, but the splitting schema is called Boosting. It begins with a single regressor and, based on the errors exceeding a specified threshold, trains additional regressors to improve predictions in those cases, thereby increasing overall accuracy (Natekin and Knoll 2013).

- Multilayer Perceptron (MLP): One of the most well-known artificial neural network architectures. It comprises multiple layers of interconnected artificial neurons connected in a feedforward schema, meaning that information flows from the input layer through hidden layers to the output layer. The MLP’s structure consists of an input layer, one or more hidden layers, and an output layer, with each layer containing multiple neurons. It is one of the most widely used techniques for pattern recognition (Taud and Mas 2018) and has a long record of successful applications.

**Table 3:** Dimensional reduction setting

Dimensional reduction	Setting
PCA	Solver = Singular value decomposition
	Number features = 162
F-score	Features = 90% of the total
MI	Number of neighbors = 3
	Features = 90% of the total

Table 4 details the configuration used for each of the regressors.

**Table 4:** Regressor setting

Regressor	Setting
PLS	Number of components = 2
	Number of iterations = 500
SGD	Loss function = ‘Squared Error’
	Penalty = L2
	Number of iterations = 1000
	Learning rate = 0.1
SVR	kernel = ‘rbf’
	Gamma = 0.7
	C = 1
	Epsilon = 0.1
Random Forest	Number of estimator = 100
	Split_criterion = Squared Error
	Max depth of the trees = unlimited
Bagging Tree Regressor	Base estimator = Decision Tree
	Number of estimator = 10,
Gradient Boosting	Number of estimator = 10
	Learning rate = 0.1
	Loss function = Squared Error
	Split criteria = Friedman’s MSE
MLP	Architecture layer = [162, 100, 1]
	Activation function = ReLU
	Max. iterations = 1000
	Batch size = 200
	Learning rate = 0.001
	Regularization = 0.001*L2
	Early stopping = True
	Validation subset = 10% of the training

All the steps and analyses carried out were performed using Python and Scikit-Learn library (Pedregosa et al. 2011). A total of 7 regressors, 4 dimensionality reduction techniques, and 3 data scaling options were used; therefore, 84 different combinations were tested. Each of these pipelines utilized the 81 transmittance values and 81 absorbance values obtained from the sensors as the input, with the output being one of the laboratory measurements, i.e., COD or TSS values.

It should be mentioned that the dimensional reduction step was exclusively applied to the signals obtained from the sensors, i.e., the input data. In contrast, the output data obtained from laboratory measurements remained unchanged throughout the analysis. This distinction ensures that the preprocessing step of dimensional reduction was focused solely on the input data, while the integrity of the output data remained preserved.

However, since data play such a fundamental role in the performance of a model, a cross-validation procedure was also carried out for each pipeline, so the results achieved are independent of the random partition of data used for their adjustment and evaluation. In this work, a tenfold cross-validation schema was used, so each pipeline was adjusted and evaluated 10 times to generate 10 test values, from which the average value was obtained. To this end, each of the 15 sub-datasets presented in Table 1 was divided into 10 equal randomly sampled partitions, and 10 different experiments were performed. In each step of the cross-validation procedure, 9 of the partitions were used for adjustment or training, while the remaining partition was used for validation of the results. Each data point is used only once for testing, but the models differ because they have been trained and adjusted with different data. The focus is on selecting the steps of the pipeline rather than on a specific model. This approach allows the pipeline to be extrapolated to other locations, while the models themselves perform based on the data they were trained on.

Therefore, a total of 840 pipelines were tested for each sub-dataset. Considering that 15 different sub-datasets were generated according to geographical location and type of water, the results therefore represent the information extracted from 12,600 models for each target pollutant, COD, and TSS. The objective of this extensive model generation was to establish a three-level classification system (Lepot et al. 2016). This system ranks the times that each regression, scaling, and dimensionality reduction technique achieves a certain position in the rankings across the different subsets of data. In addition, this ranking was performed taking into account the type of wastewater studied, differentiating between raw and treated wastewater.

**Fig. 2:**
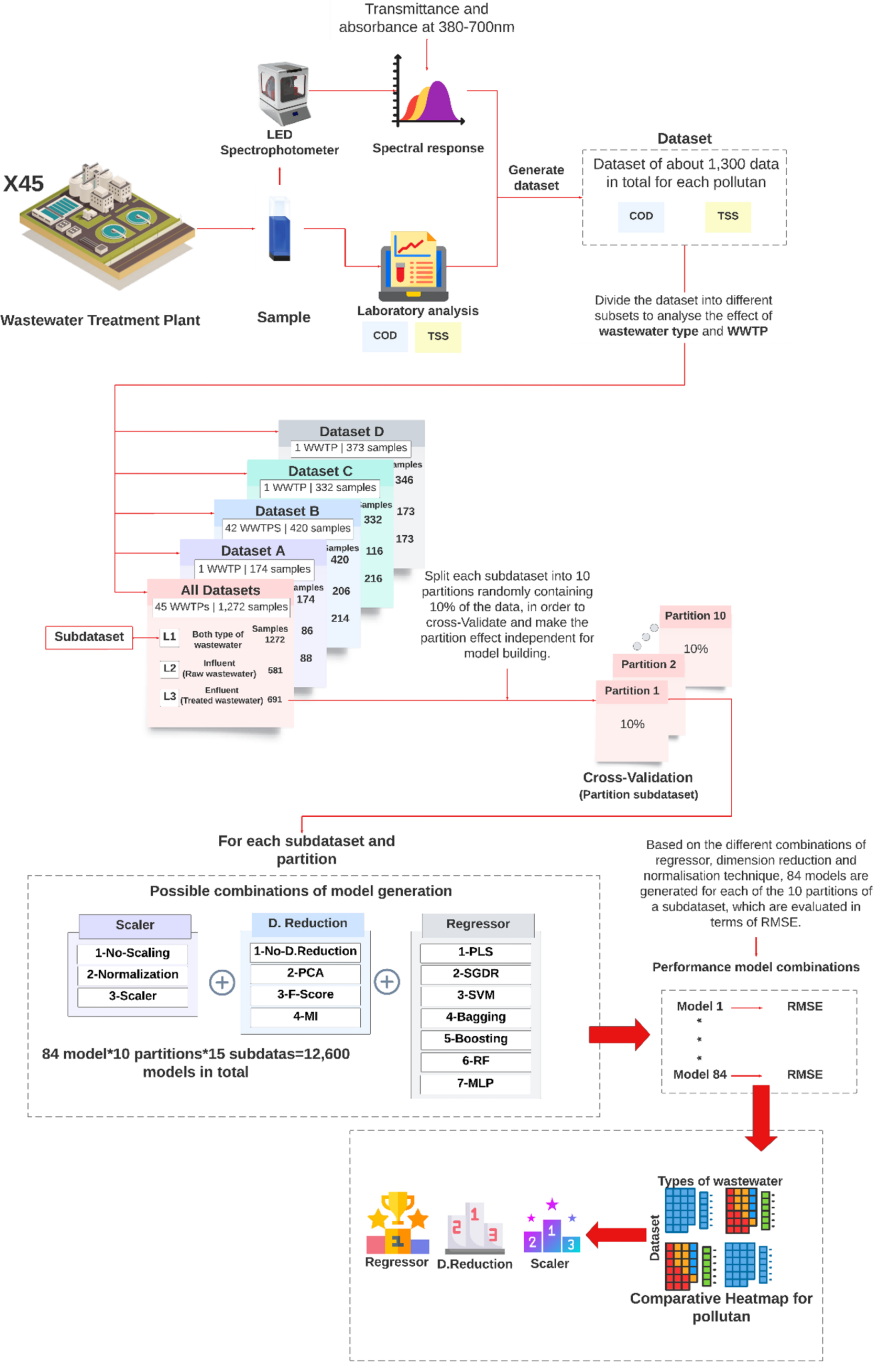
Diagram of the analysis process carried out in this research work

To clarify the process carried out, Fig. 2 shows a diagram of the pipeline followed to determine which regressor, scaling, and dimensionality reduction provided the best results with each dataset for raw and treated wastewater samples, both jointly and individually. For clarity in the exposition of the process, the diagram in Fig. 2 only shows the process for a single partition of only one of the sub-datasets, when in practice it was carried out on all the partitions of all the sub-datasets under study.

Thus, and as can be seen in Fig. 2, over 1300 wastewater samples were collected from different wastewater treatment plants (WWTPs) using LED spectrophotometry (Carreres-Prieto, et al 2019*)* to obtain spectral response data and laboratory analytical values for COD and TSS. As a result, five datasets were generated for each pollutant: four corresponding to data from a single WWTP or from a localized set of them (datasets A–D*)*, as well as an additional dataset that combined all the previous ones together (all datasets). Subsequently, a cross-validation process was performed with a tenfold partitioning scheme, applying 84 different models per partition, resulting in a total of 12,600 models trained and evaluated across 15 sub-datasets. The mean and standard deviation of each cross-validation were calculated to compare pipeline configurations. Based on the median values of the combinations of regression, dimensionality reduction, and scaling techniques, a comparative heat map was produced in matrix form, ranking the types of wastewater and data sets worked on according to the frequency with which each configuration achieved a top three position in performance.

In order to obtain a general analysis from the heatmap information (one in terms of COD and the other in terms of TSS), where the number of times that a regression, scaling, or dimensionality reduction technique occupied a certain position in the ranking was determined, expressed as a percentage of the times that it occupied a certain position with respect to the set, a ranking for all the study datasets and segregated by type of water was established. This was based on the sum of the number of times that a given regressor, dimensionality reduction technique, and scaling occupied a given position for all the study datasets, in order to determine the most appropriate one to apply for each type of water (or combination thereof) regardless of where the data come from, i.e., regardless of the WWTP or geographic area.

## Results and discussion

### Analysis of datasets

**Fig. 3:**
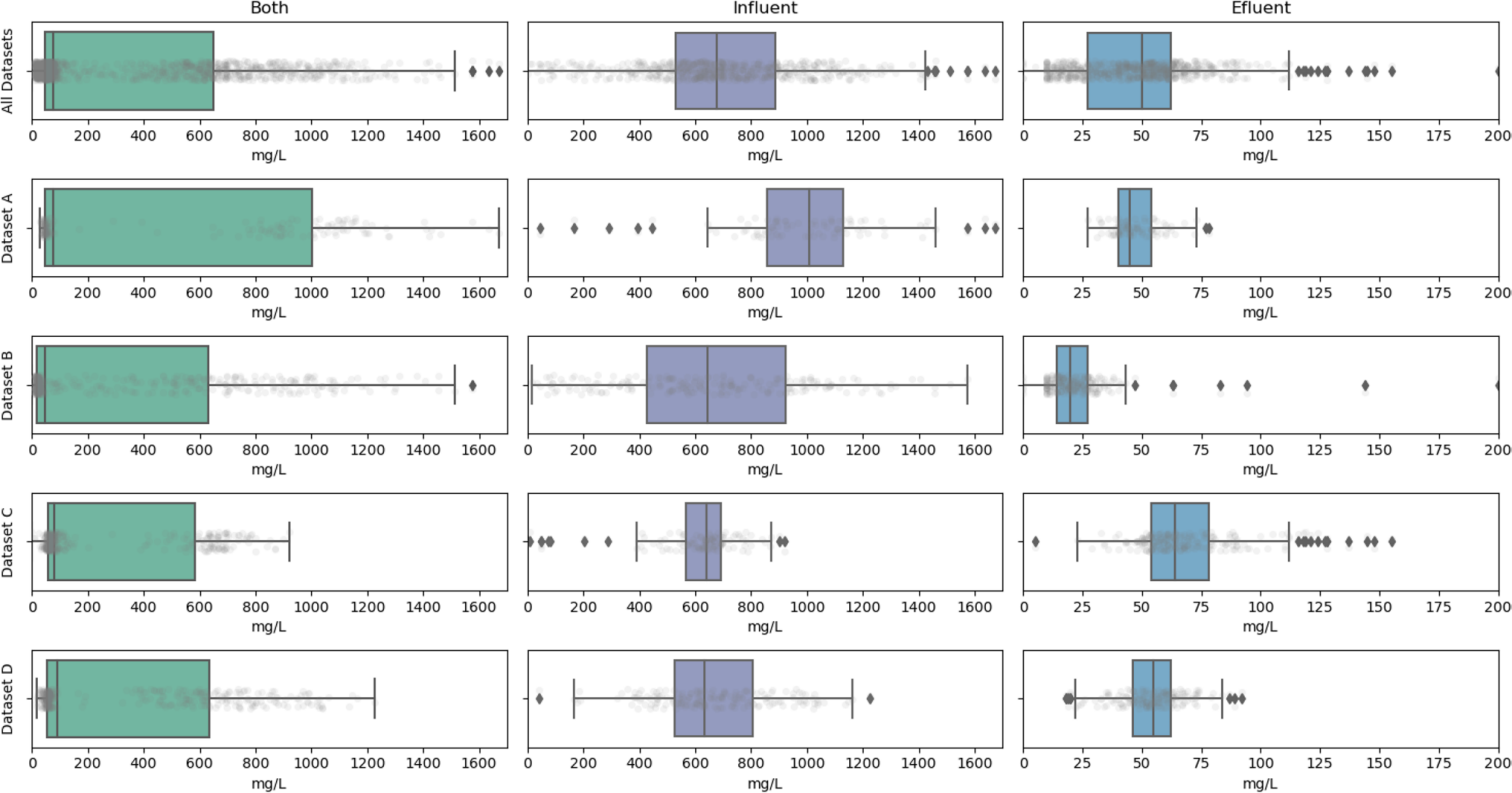
COD data distribution diagram by dataset

**Fig. 4:**
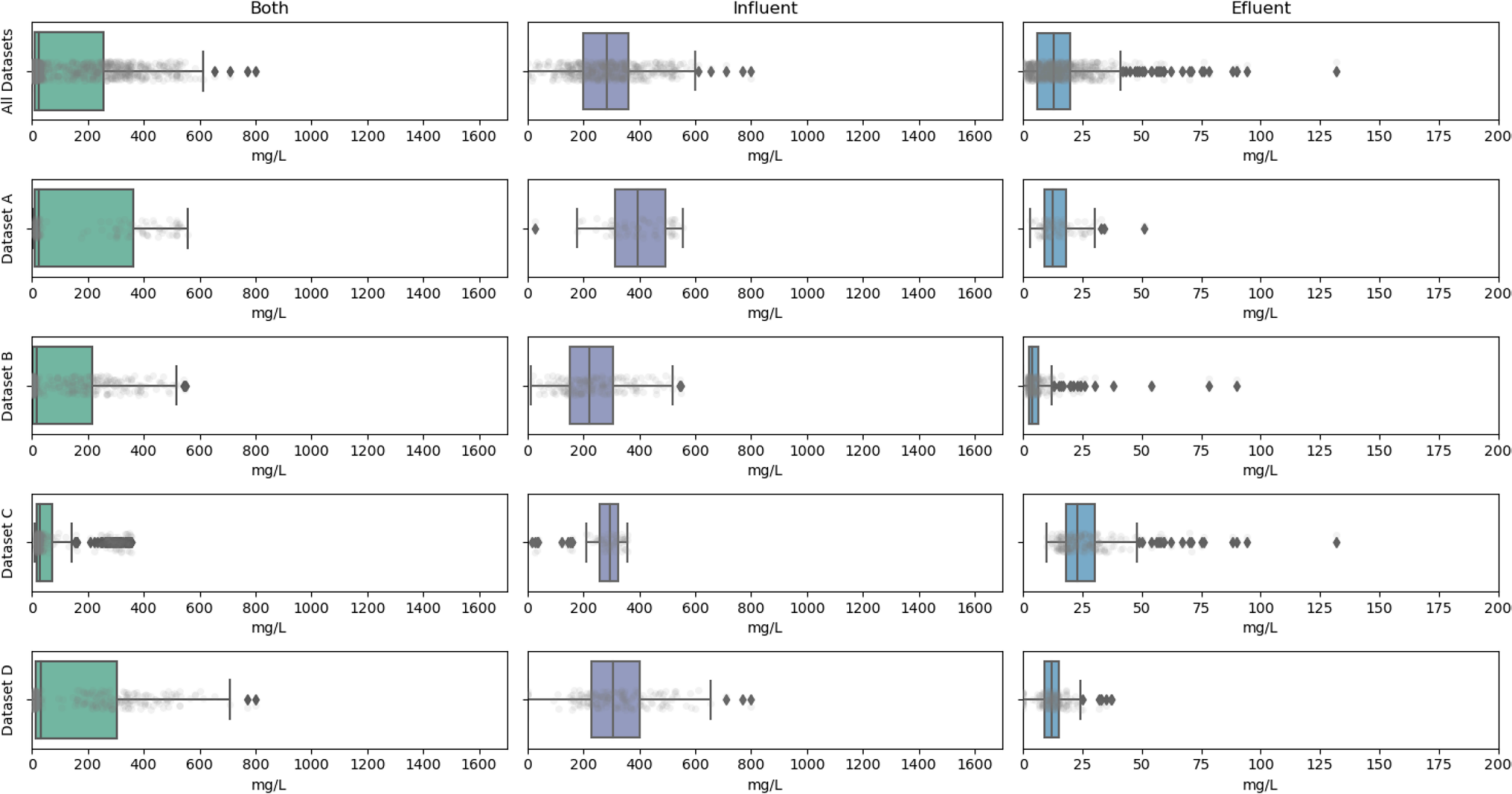
TSS data distribution diagram by dataset

The distribution of the data in terms of COD and TSS associated with each of the study sub-datasets, i.e., by dataset and wastewater type: effluent, influent, or both, are shown in Figs. 3 and 4, using box-and-whisker plots.

As expected, the overall distribution of COD and TSS records presented a bimodal distribution. Thus, effluent pollutants showed a very narrow range of variation with threshold values ideally below EU environmental legislation limits. On the other hand, influent records showed far greater variability, due to the differences in the origin of the wastewater, produced at different geographical locations, and to a lesser extent due to the daily and seasonal variation patterns produced internally in each catchment area. This data clusterization will have some implications when performing model adjustments, as will be discussed later.

Furthermore, the datasets also presented some statistical outliers which have been maintained in some of the datasets, to enable the conditions for analyzing and understanding the variability inherent in the real data to be considered and to develop models that more accurately adapt to the complexity and natural fluctuations of the phenomena studied. Data which, in the opinion of an expert, undeniably correspond to analytical failures in the WWTP laboratories were eliminated.

### Benchmark chemical oxygen demand

In this section, the impact of factors such as regressor type, scaling techniques, and dimensionality reduction techniques on the performance of characterization models was examined using a cross-validation methodology on each sub-dataset. The same data partitions were used to analyze each technique on each sub-dataset.

**Fig. 5:**
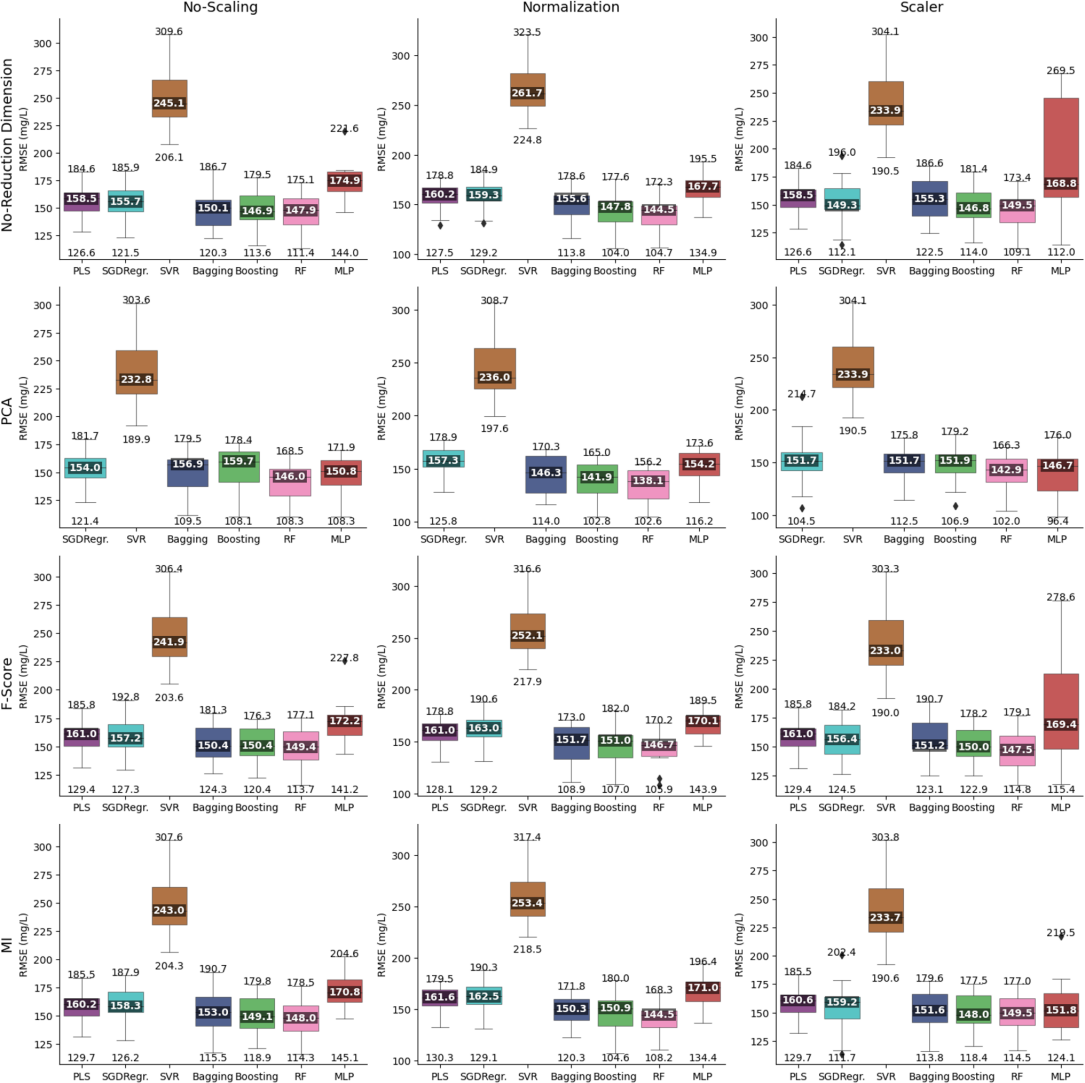
Comparative box-and-whisker plot, organized by dimension reduction and scaling, for each of the study regressors, for the dataset that included all water types and WWTPs (sub-dataset L1) for COD

The results obtained from the different regressors as a function of dimensionality reduction methods (organized by rows) and scaling techniques (arranged in columns) in terms of COD are shown in Fig. 5 using a matrix structure. For clarity, the figure only presents the results related to sub-dataset L1 of Table 1, which encompasses all types of water and WWTPs analyzed, with the results for the other datasets being given in the Supplementary Information.

As can be observed, the median was taken as the measure of the central trend for each of the partitions, in order to mitigate the effect of outlier partitions (those providing the best and worst performances), thereby ensuring that the results presented better represented the overall performance of the combinations studied.

The performance of each of the regressors, dimensionality reduction, and scaling technique as a function of the type of wastewater under study (raw wastewater, treated wastewater, or a combination of both) and the working datasets (including the one covering all WWTPs under study) are presented in matrix form in Fig. 5. Within each cell of this matrix (representing one of the sub-datasets in Table 1), three heatmaps are presented, in terms of minimizing the root mean square error (RMSE). Figure S1 in the Supplementary Information provides the results of this analysis in terms of *R*^2^. The values shown in the heatmaps indicate the percentage of times that a given combination occupies a given position in the ranking in terms of RMSE minimization.

Each heatmap focuses on one of the three key aspects of the analysis: regressors, dimensionality reduction, and scaling, and establishes a ranking, in terms of how many times each combination ranked first, second, or third in terms of performance. This ranking provides an intuitive view of trends and patterns in the data, facilitating the identification of the most effective combinations for each specific scenario.

**Fig. 6:**
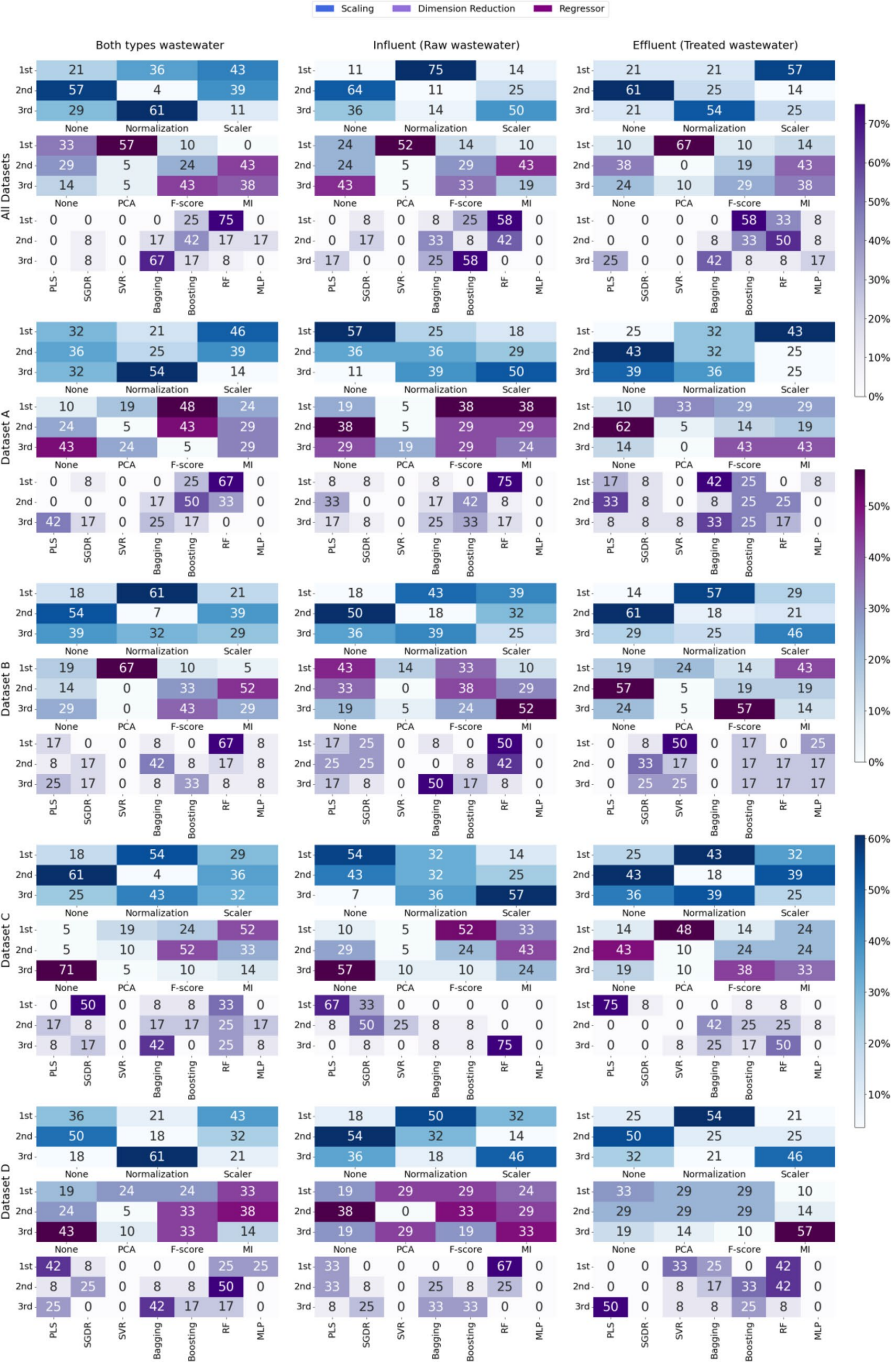
Comparative performance matrix by dataset and water type, broken down by dimension reduction, scaling, and regressor, evaluated by RMSE, for COD

As can be seen in Fig. 6, in the combination of all the datasets, the RF regressor stood out, leading with a notable frequency, reaching 75% in the global analysis. The dimension reduction technique PCA proved to be the most effective, dominating in 57% of the cases at the global level, while its relevance rose to 67% in specific treated water samples. Scaler normalization was preferred in 43% of the cases globally, although for raw water samples specifically, standard normalization emerged as the predominant option with 75%.

Detailing the results by dataset, it must be highlighted that in dataset A, RF led in 67% of the cases when combining all types of water, followed by the Boosting technique with 25%. For dimension reduction, F-score and MI alternated as leaders, depending on the specific context, while PCA took the lead with 33% in treated water samples. In terms of normalization, Scaler was shown to be the most successful option in most scenarios, highlighting that the absence of normalization provided the best results 57% of the times for raw wastewater.

Datasets B to D reflected similar patterns, with RF maintaining a solid lead in raw and combined water scenarios, reaching 67% and 50%, respectively, in some contexts. SGDR and PLS emerged as the leading regressors in specific situations, aspect observed in works such as Lepot et al. (2016), where PLS stands out in first position in the ranking., with SGDR leading in 50% of cases for raw and treated water combinations in dataset C. Variability in the choice of dimension reduction and normalization techniques highlighted the adaptability needed depending on the sample type, with MI dominating at 52% in certain contexts and simple normalization leading at 54% in others.

To complement the detailed analysis presented in Fig. 6, Table 5 consolidates and synthesizes key information on the effectiveness of the different dimensionality reduction, scaling, and regressor configurations. That table focuses on the accumulation of ranking positions based on minimizing RMSE, providing a quantitative perspective on how often each approach performed outstandingly well on the different data sets and water types analyzed, so the study variable (regressor, scaling, or dimensionality reduction) that occupied the 1st position, for a given wastewater type, a greater number of times indicated a higher suitability for treating and analyzing that specific type of wastewater.

**Table 5:** Summary of ranking frequencies for each regressor technique (A), dimensionality reduction (B), scaling (C) by RMSE for COD

**RMSE**									
	**Both**			**Influent**			**Effluent**		
	**1º**	**2º**	**3º**	**1º**	**2º**	**3º**	**1º**	**2º**	**3º**
**PLS**	1	0	1	1	1	0	1	1	1
**SGDR**	1	0	0	1	1	0	0	1	1
**SVM**	0	0	0	0	0	0	1	0	1
**Bagging**	0	1	3	0	0	2	1	1	2
**Boosting**	0	2	1	0	1	3	0	1	0
**RF**	**3**	2	0	**4**	2	1	**2**	1	1
**MLP**	0	0	0	0	0	0	0	0	0
**(A)**									
	**Both**			**Influent**			**Effluent**		
	**1º**	**2º**	**3º**	**1º**	**2º**	**3º**	**1º**	**2º**	**3º**
**No-D.Reduc.**^**1**^	0	0	3	1	2	3	1	4	0
**PCA**	**2**	0	0	2	0	0	**3**	1	0
**F-score**	1	2	2	**3**	1	1	0	1	3
**MI**	**2**	3	0	1	2	2	1	1	3
**(B)**									
	**Both**			**Influent**			**Effluent**		
	**1º**	**2º**	**3º**	**1º**	**2º**	**3º**	**1º**	**2º**	**3º**
**No-scaling**	0	4	1	2	5	0	0	5	1
**Normal.**^**2**^	2	0	4	**3**	1	1	**3**	0	2
**Scaler**	**3**	1	0	0	0	4	2	0	2
**(C)**									

^1^ No-Dimension Reduction applied

^2^ Normalization

The analysis relating to the type of regressor employed based on the water type is shown in Table 5 part A. When combining all types of wastewaters, Random Forest stood out, ranking first in three out of the five datasets analyzed. This was closely followed by PLS and SGDR, although the latter two only demonstrated a notable performance in minimizing RMSE in one single dataset each, specifically datasets D and C, respectively. Random Forest and Boosting tied in the second position with two datasets each.

When considering raw wastewater exclusively, the superiority of Random Forest was once again highlighted, leading in the first position in four out of the five datasets studied. In contrast, PLS only achieved the top position in dataset C. However, in terms of the second position, there was a greater variety of regressors observed, with Random Forest still leading, followed by Boosting, PLS, and SGDR.

Regarding the specific analysis of treated water, it was observed that Random Forest remained the most effective regressor, closely followed by PLS, SVM, and Bagging, leading in datasets C and B.

In relation to the dimensionality reduction technique (Table 5 part B), its effectiveness varied depending on the type of wastewater considered. When all types of wastewaters were combined, PCA and MI each led in two datasets in terms of reducing the RMSE, followed by the F-score. However, MI stood out in the second position of the ranking, followed by the F-score.

For raw wastewater samples, the F-score enabled the greatest reduction in RMSE in three of the datasets, followed by PCA in two, while MI and the absence of dimensionality reduction techniques led in only one dataset each. It should be noted that in certain datasets, there was a tie between two techniques, as in the case of dataset A, where there was a tie between F-score and MI, and in dataset D between PCA and F-score. On the other hand, the second position in the ranking was led by MI and the absence of dimensionality reduction techniques.

As for treated wastewater samples, PCA led in three datasets, followed by the absence of dimensionality reduction techniques and MI, albeit to a lesser extent. In contrast, the second position in the ranking was led by the absence of dimensionality reduction techniques, presenting the greatest reductions in RMSE in four datasets.

In terms of the normalization techniques applied to the data, interesting patterns emerged across different contexts of wastewater samples. In datasets combining both raw and treated wastewater samples, Scaler ranked first in three datasets, followed by normalization in two. However, it should be remarked that in the second position of the ranking, not applying any normalization technique occupied the top spot.

In the specific case of the analysis of raw wastewater samples, normalization stood out, taking the first position in three of the datasets studied. Interestingly, the second position in the ranking was again dominated by the decision not to apply any normalization technique.

On the other hand, when focusing on treated wastewater samples, it is evident that the application of data normalization yielded the best results in terms of minimizing the RMSE, followed by scaling (Scaler). However, in accordance with previous findings, the second position in the ranking was led by the choice of not applying any normalization technique.

### Benchmark total suspended solids

**Fig. 7:**
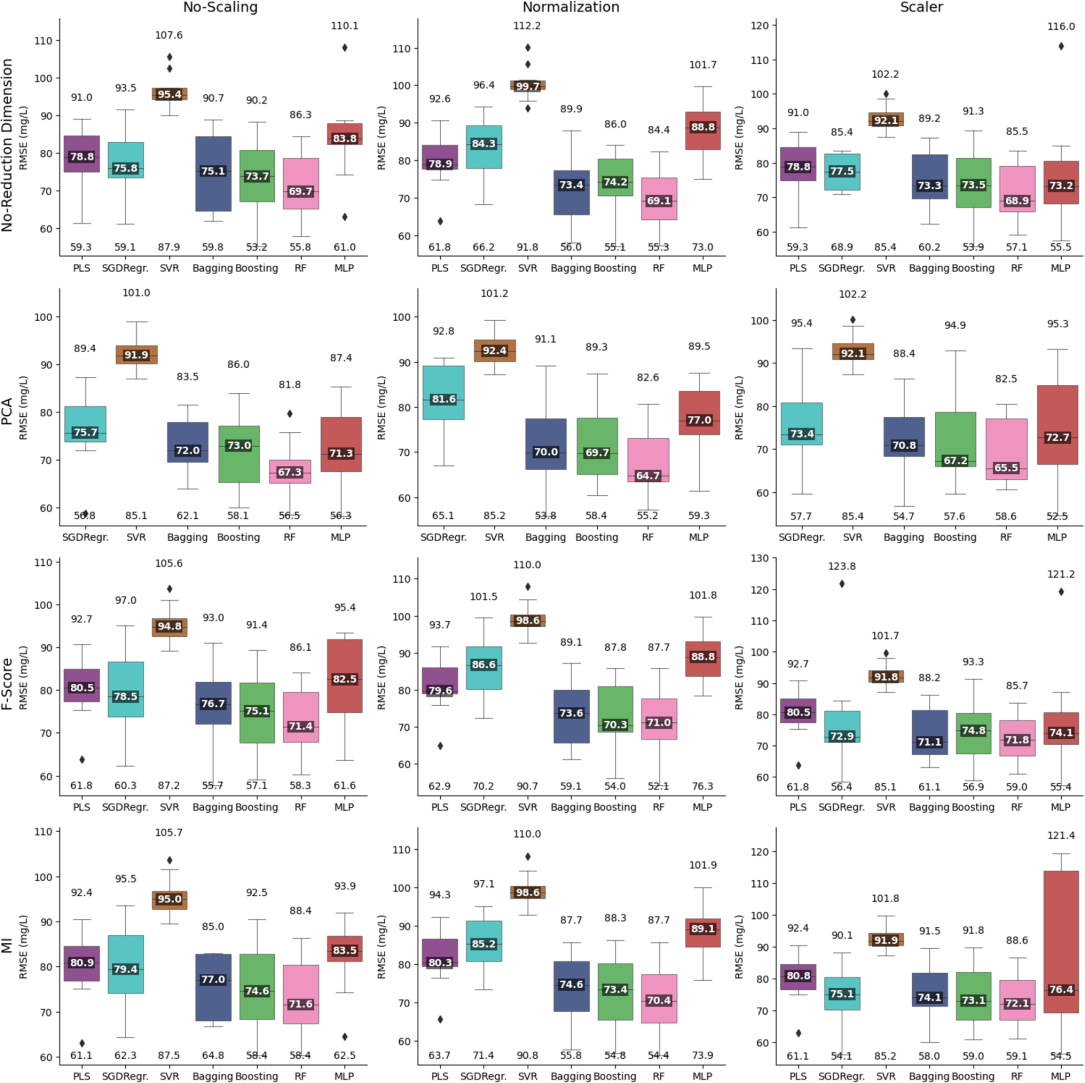
Comparative box-and-whisker plot, organized by dimension reduction and scaling, for each of the study regressors, relative to the dataset including all water types and WWTPs (sub-dataset L1) for TSS

The performance of the models for each of the 10 partitions, organized by type of scaling technique (columns) and dimensionality reduction technique (rows), is shown in Fig. 7 for sub-dataset L1 of Table 2 (which collects all WWTPs and wastewater types). The Supplementary Information contains the analysis for the rest of the sub-datasets.

As can be seen, the median RMSE did not vary greatly between the different types of scaling and dimensionality reduction.

**Fig. 8:**
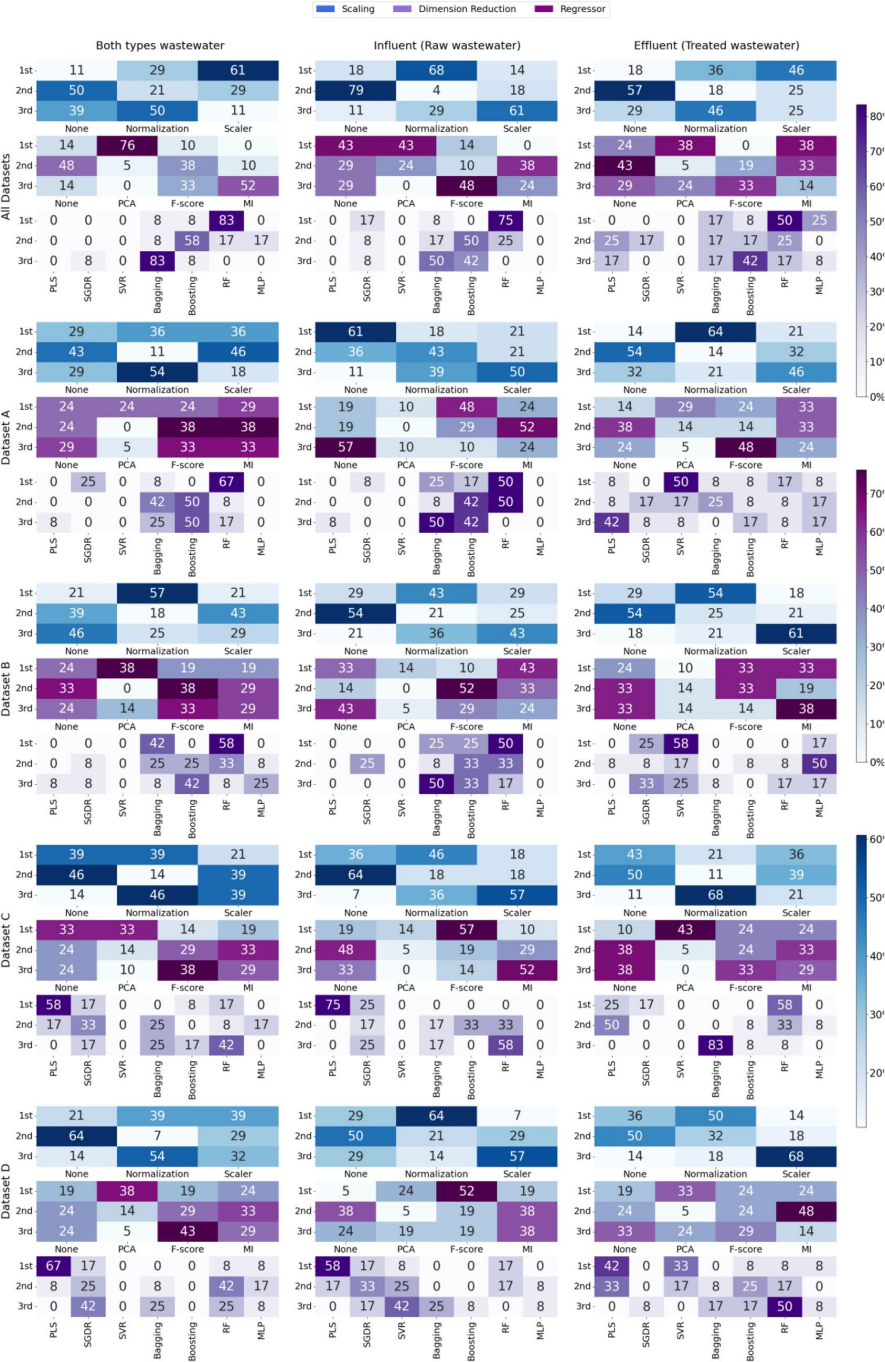
Comparative performance matrix by dataset and water type, broken down by dimension reduction, scaling and regressor, evaluated by RMSE, for TSS

Regarding the performance of the different combinations of regressor, scaling, and dimension reduction techniques, in terms of TSS characterization, a comparative matrix analysis for each type of water and dataset, in terms of minimizing RMSE, is shown in Fig. 8. Figure S16 in the Supplementary Information details the results of this analysis in terms of *R*^2^.

Upon analyzing the effect of each of the regression, dimension reduction, and normalization techniques on each of the study datasets, it was observed that in the case of combining all the datasets and types of wastewater, the RF regressor appeared as dominant, in the first position 83% of the times. The PCA dimension reduction technique excelled by securing the first position in 76% of the cases. In terms of data normalization, Scaler stood out significantly. Within the specific analysis by sample type, when focusing on raw water, RF remained the preferred regressor, leading 75% of the time, although diversification was observed in dimension reduction with a tie between no dimension reduction and PCA, each with 43%. For normalization, the standard technique clearly prevailed and was the most used in 68% of the cases. On the other hand, in treated water samples, RF was less predominant, at 50%, and in dimension reduction, PCA and MI tied at 38%, which reflects the existence of several effective approaches. Scaler normalization was the most frequent, with a 46% prevalence.

In dataset A, RF led 67% of the times in an overall analysis, with a moderate preference for MI in dimension reduction (29%) and a tie between standard normalization and Scaler (36%). In dataset B, RF stood out as the most effective regressor (58%), PCA dominated in dimension reduction (38%), and standard normalization was the predominant technique (57%). Dataset C showed PLS as the main regressor (58%), with a remarkable diversity in dimension reduction and normalization techniques, with no technique emerging as common in certain contexts. Finally, in dataset D, PLS again led as a regressor (67%), with PCA as the most common choice for dimension reduction (38%) and a balance between standard and Scaler normalization techniques (39%).

Table 6 summarizes the information shown in Fig. 8 regarding the cumulative accumulated value of the ranking positions based on the RMSE metrics as a function of the regressor, scaling, and dimension reduction technique employed.

**Table 6:** Summary of ranking frequencies for each regressor technique (A), dimensionality reduction (B), scaling (C) by RMSE for TSS

**RMSE**									
	**Both**			**Influent**			**Effluent**		
	**1º**	**2º**	**3º**	**1º**	**2º**	**3º**	**1º**	**2º**	**3º**
**PLS**	2	0	0	2	0	0	1	2	1
**SGDR**	0	1	1	0	1	0	0	0	1
**SVM**	0	0	0	0	0	1	**2**	0	0
**Bagging**	0	0	1	0	0	3	0	1	2
**Boosting**	0	2	2	0	3	0	0	1	0
**RF**	**3**	2	1	**3**	3	1	**2**	0	1
**MLP**	0	0	0	0	0	0	0	1	0
**(A)**									
	**Both**			**Influent**			**Effluent**		
	**1º**	**2º**	**3º**	**1º**	**2º**	**3º**	**1º**	**2º**	**3º**
**No-D.Reduc.**^**1**^	1	1	0	1	2	2	0	4	2
**PCA**	**4**	0	0	1	0	0	**3**	0	0
**F-score**	0	2	4	**3**	1	1	1	1	2
**MI**	1	3	2	1	3	2	**3**	1	1
**(B)**									
	**Both**			**Influent**			**Effluent**		
	**1º**	**2º**	**3º**	**1º**	**2º**	**3º**	**1º**	**2º**	**3º**
**No-Scaling**	1	3	1	1	4	0	1	5	0
**Normal.**^**2**^	**4**	0	4	**4**	1	0	**3**	0	2
**Scaler**	3	2	0	0	0	5	1	0	3
**(C)**									

^1^ No-Dimension Reduction applied

^2^ Normalization

In Table 6 part A, dedicated to analyzing the performance of different regressors, Random Forest stands out as the algorithm with the best performance both in the configuration that included both types of wastewater (both) and in the influent. However, for the effluent, RF and SVM shared the leading position, leading in the same number of datasets. This performance pattern was also reflected in the *R*^2^ values, indicating consistency in the results obtained.

Regarding the size reduction techniques (Table 6 part B), PCA ranked first when considering all types of wastewater combined. On the other hand, in the specific analysis of the influent (raw wastewater), the F-score showed a superior performance, occupying the first position. For effluent, a tie was observed between PCA and MI in terms of leadership in the same number of cases. The second position varied depending on the type of water analyzed, where in combined wastewater, MI and F-score demonstrated outstanding performance, while for influent, MI excelled followed by the option of not applying dimension reduction.

Table 6 part C shows that data normalization significantly improved the results in all the contexts analyzed, positioning it as the most effective strategy for each type of wastewater. This underscores the importance of data normalization as a preliminary step in model performance analysis.

## Conclusions

LED spectrophotometry has proven to be an effective technique for the indirect characterization of the contaminant load in wastewater, obviating the need for pretreatment or chemical reagents by correlation models. The construction of these models, in terms of the regressor selected, the type of scaling, and the pretreatment method applied, is key to ensure their accuracy and validity in different wastewater contexts, influenced by variables such as population demographics, the presence of industry, and climate conditions.

This study evaluated the influence of seven regression techniques, three scaling strategies, and four dimensionality reduction methods on 1300 raw and treated wastewater samples from 45 treatment plants in different regions of Spain and Santiago of Chile, over 4 years.

The RMSE was used to analyze the performance of the models as it has been established as the best indicator of performance, since the *R*^2^ is not always reliable, especially because it does not consider the scale of the data and underestimates the performance in the face of non-linear or complex relationships.

The analyses indicated that the Random Forest model excelled in the characterization of COD, proving to be the most suitable algorithm due to its high efficiency, evidenced in 75% of the cases analyzed. Furthermore, the importance of a meticulous selection of preprocessing techniques, especially normalization and dimensionality reduction, was highlighted in order to adapt to the specific characteristics of each water sample.

Regarding the characterization of TSS by spectrophotometric analysis, RF emerged as the most efficient regressor, predominating in 83% of the cases. The PCA dimensionality reduction technique proved its relevance and was the most effective in 76% of the situations. This study underscores the need for adequate data preprocessing, with the Scaler method as the most recurrent normalization option.

Overall, Random Forrest models obtained the best performance the 75% and 83% of the model-assessed combinations for COD and TSS, respectively. In the work of Lepot et al. (2016), a small number of regression techniques were assessed for a dataset of UV–vis spectrometry data. In Lepot et al. study, PLS (partial least squares) and SVM (support vector machine) techniques occupy the top positions in the rankings. This finding is consistent with the results obtained in our research work, where PLS and SVM tend to occupy the second positions in certain datasets (datasets B to D), although with a lower performance than the RF regressor, which was not considered in the Lepot et al. study.

Significant advances in wastewater treatment process modeling have been achieved by integrating a large data set from different WWTPs and wastewater samples. This approach has enabled the development of robust and generalizable models that surpass previous studies, offering valuable tools for the optimization of treatment plants under several operational and geographical conditions.

This research work has found the effectiveness of LED spectrophotometry, in combination with advanced modeling and data analysis techniques, in the indirect characterization of wastewater pollutant load. The findings highlight the importance of properly selecting the regressor, scaling method, and dimensionality reduction techniques to maximize the accuracy and applicability of models in different wastewater contexts. The adaptability and robustness of these models open new possibilities for efficient and sustainable wastewater management, enabling more advanced monitoring.

The use of LED spectrophotometry in combination with a trained characterization model for the detection of important pollutants present in wastewater, such as COD or TSS, allows for real-time monitoring of water quality at a low cost, enabling rapid incident detection, and swift action by plant personnel, which is impossible with laboratory analysis.

## Electronic supplementary material

Below is the link to the electronic supplementary material.

Supplementary file1 (DOCX 17280 KB)

## Data Availability

Not applicable.

## Appendix

**Table 7:** Wastewater treatment plants used during the study, organized by dataset

				Population		Capacity (m3/y)		TSS			COD			BOD_5_		
Dataset		WWTPs include	Province	Served	Equivalent	Design (m3/a)	Current (m3/a)	In (mg/l)	Out (mg/l)	Perf (%)	In (mg/l)	Out (mg/l)	Perf (%)	In (mg/l)	Out (mg/l)	Perf (%)
Dataset A	1	Cabezo Beaza	Murcia	176,223	173,924	12,775,000	9,031,284	470	18	96.2	924	52	94.4	422	12	97.2
Dataset B	1	Abanilla	Murcia	3626	15,711	547,500	779,051	294	4	98.6	739	18	97.6	442	4	99.1
	2	Abarán	Murcia	13,371	12,626	1,642,500	726,065	257	5	98.1	596	28	95.3	381	3	99.2
	3	Albudeite	Murcia	1296	1043	365,000	45,738	205	8	96.1	756	31	95.9	499	4	99.2
	4	Alcantarilla	Murcia	41,447	62,342	4,745,000	2,588,649	301	6	98.0	835	33	96.0	527	4	99.2
	5	Alguazas	Murcia	9102	37,629	5,475,000	1,076,650	371	4	98.9	1208	22	98.2	765	3	99.6
	6	Archena	Murcia	24,413	54,425	2,737,500	1,792,326	459	6	98.7	1104	27	97.6	665	3	99.5
	7	Baños y Mendigo	Murcia	218	344	173,375	21,521	352	10	97.2	591	36	93.9	350	3	98.9
	8	Barinas	Murcia	756	1982	197,100	73,884	390	4	99.0	906	21	97.7	588	4	99.3
	9	Barqueros	Murcia	1030	1872	109,500	60,376	443	17	96.2	1245	58	95.3	679	6	99.1
	10	Beniel Nueva	Murcia	11,900	25,818	1,825,000	1,245,618	659	4	99.4	944	26	97.2	454	3	99.3
	11	Blanca	Murcia	5184	5636	730,000	356,464	271	4	98.5	559	19	96.6	346	3	99.1
	12	Cabezo de la Plata	Murcia	104	358	44,165	44,165	248	11	95.6	1024	30	97.1	702	3	99.6
	13	Calasparra	Murcia	9505	26,938	2,190,000	659,778	408	3	99.3	1426	23	98.4	894	3	99.7
	14	Campos del Río	Murcia	1998	1635	547,500	88,252	212	5	97.6	649	21	96.8	406	3	99.3
	15	Cañada de la leña	Murcia	93	28	21,900	6166	68	19	72.1	172	56	67.4	99	6	93.9
	16	Cañares / Bronchos	Murcia	442	195	1,350,500	54,371	805	2	99.7	3253	2751	37.5	156	3	98.0
	17	Casas Nuevas	Murcia	152	220	73,000	8170	847	6	99.3	1,190	28	97.6	590	4	99.3
	18	Ceutí Nueva	Murcia	11,774	36,311	2,920,000	1,052,685	448	11	97.5	1,274	33	97.4	755	3	99.6
	19	Cieza	Murcia	33,797	63,567	3,650,000	2,485,914	362	5	98.6	872	23	97.4	560	3	99.5
	20	Corvera	Murcia	2443	2464	109,500	133,534	284	2	99.3	682	24	96.5	404	3	99.3
	21	El Cantón	Murcia	66	506	18,250	18,250	324	18	94.4	1058	34	96.8	608	5	99.2
	22	El Raal	Murcia	15,940	23,706	2,737,500	3,950,557	151	7	95.4	240	21	91.3	131	4	96.9
	23	El Valle	Murcia	194	464	511,000	58,089	378	5	98.7	343	17	95.0	175	3	98.3
	24	Fortuna	Murcia	7557	11,544	912,500	423,126	445	9	98.0	975	34	96.5	598	4	99.3
	25	Fuente Librilla	Murcia	579	1418	146,000	44,776	266	19	92.9	1122	38	96.6	694	4	99.4
	26	Hacienda Riquelme	Murcia	224	658	574,875	64,366	145	5	96.6	313	24	92.3	159	3	98.1
	27	Jumilla Nueva	Murcia	24,588	70,595	4,380,000	1,739,564	825	3	99.6	1761	24	98.6	889	3	99.7
	28	La Murta	Murcia	91	545	44,165	15,378	353	4	98.9	1271	28	97.8	776	3	99.6
	29	Lorqui	Murcia	6622	26,108	1,825,000	1,221,497	376	4	98.9	835	19	97.7	468	3	99.4
	30	Macisvenda	Murcia	504	557	41,975	26,219	242	5	97.9	730	30	95.9	465	3	99.4
	31	Molina Norte	Murcia	68,296	218,823	9,125,000	6,093,740	490	6	98.8	1456	36	97.5	786	3	99.6
	32	Mosa Trajectum	Murcia	144	285	642,400	42,568	177	3	98.3	270	15	94.4	147	3	98.0
	33	Mula Nueva	Murcia	15,496	17,210	2,190,000	672,031	335	2	99.4	892	18	98.0	561	3	99.5
	34	Murcia Este	Murcia	375,775	553,451	36,500,000	36,952,999	277	9	96.8	577	32	94.5	328	5	98.5
	35	Pliego	Murcia	3631	4490	547,500	162,769	583	3	99.5	1150	23	98.0	604	3	99.5
	36	Pol. Ind. Fortuna	Murcia	0	584	65,700	22,945	797	30	96.2	855	68	92.0	557	13	97.7
	37	Santomera Norte	Murcia	14,956	16,139	2,190,000	1,137,404	242	5	97.9	526	33	93.7	311	4	98.7
	38	Sucina Nueva	Murcia	1924	3,634	1,825,000	173,650	212	3	98.6	681	22	96.8	458	3	99.3
	39	Torres de Cotillas N	Murcia	19,996	53,597	4,380,000	1,602,051	641	9	98.6	1281	21	98.4	733	3	99.6
	40	El Trampolín	Murcia	149	158	73,000	13,930	329	37	88.8	396	33	91.7	248	4	98.4
	41	Yecla	Murcia	31,876	43,586	2,920,000	1,648,354	490	7	98.6	1015	20	98.0	579	3	99.5
	42	Yecla Raspay	Murcia	97	109	18,250	8385	147	5	96.6	477	18	96.2	286	4	98.6
Dataset C	1	Mapocho–Treval	Chile	2,807,000	3,674,880	-	760,320	209	23	89	696	64	91	-	-	-
Dataset D	1	Bens WWP	Galicia	374.304	774.433	47.808.576	46.964.352	330,20	12,48	95,54	635,20	55,57	90,15	-	-	-
