# Supplementary material for: Optimization of indirect wastewater characterization using led spectrophotometry: a comparative analysis of regression, scaling, and dimensionality reduction methods
Source: Environ Sci Pollut Res Int. 2024 Aug 28;31(42):54481–501. doi: 10.1007/s11356-024-34714-8 (PMC11413097; doi:10.1007/s11356-024-34714-8)
Supplement: Supplementary file 1 — Supplementary file1 (DOCX 17280 KB) [file 11356_2024_34714_MOESM1_ESM.docx]

*Supplementary Materials*

**OPTIMIZATION OF INDIRECT WASTEWATER CHARACTERIZATION USING LED SPECTROPHOTOMETRY: A COMPARATIVE ANALYSIS OF REGRESSION, SCALING, AND DIMENSIONALITY REDUCTION METHODS**

Daniel Carreres-Prieto^1*^, Enrique Fernandez-Blanco^2^, Daniel Rivero^2^, Juan R. Rabuñal^3^,Jose Anta^4^, Juan T. García^5^

1. Department of Engineering and Applied Techniques, Centro Universitario de la Defensa de San Javier, 30720 Santiago de la Ribera, Spain
2. Department of Computer Science and Information Technologies, Universidade da Coruña, CITIC, 15071 A Coruña, Spain
3. Artificial Neural Networks and Adaptative Systems Research Group (RNASA) and Centre of Technological Innovation in Construction and Civil Engineering (CITEEC), University of A Coruña, 15071 A Coruña, Spain
4. Water and Environmental Engineering Research Team (GEAMA), Universidade da Coruña, CITEEC, Civil Engineering School, 15071, A Coruña, Spain
5. Department of Mining and Civil Engineering, Universidad Politécnica de Cartagena, 30202 Cartagena, Spain

* Correspondence: daniel.carreres@cud.upct.es (D.C.-P.);

**3.1. Chemical Oxygen Demand (COD)**

| **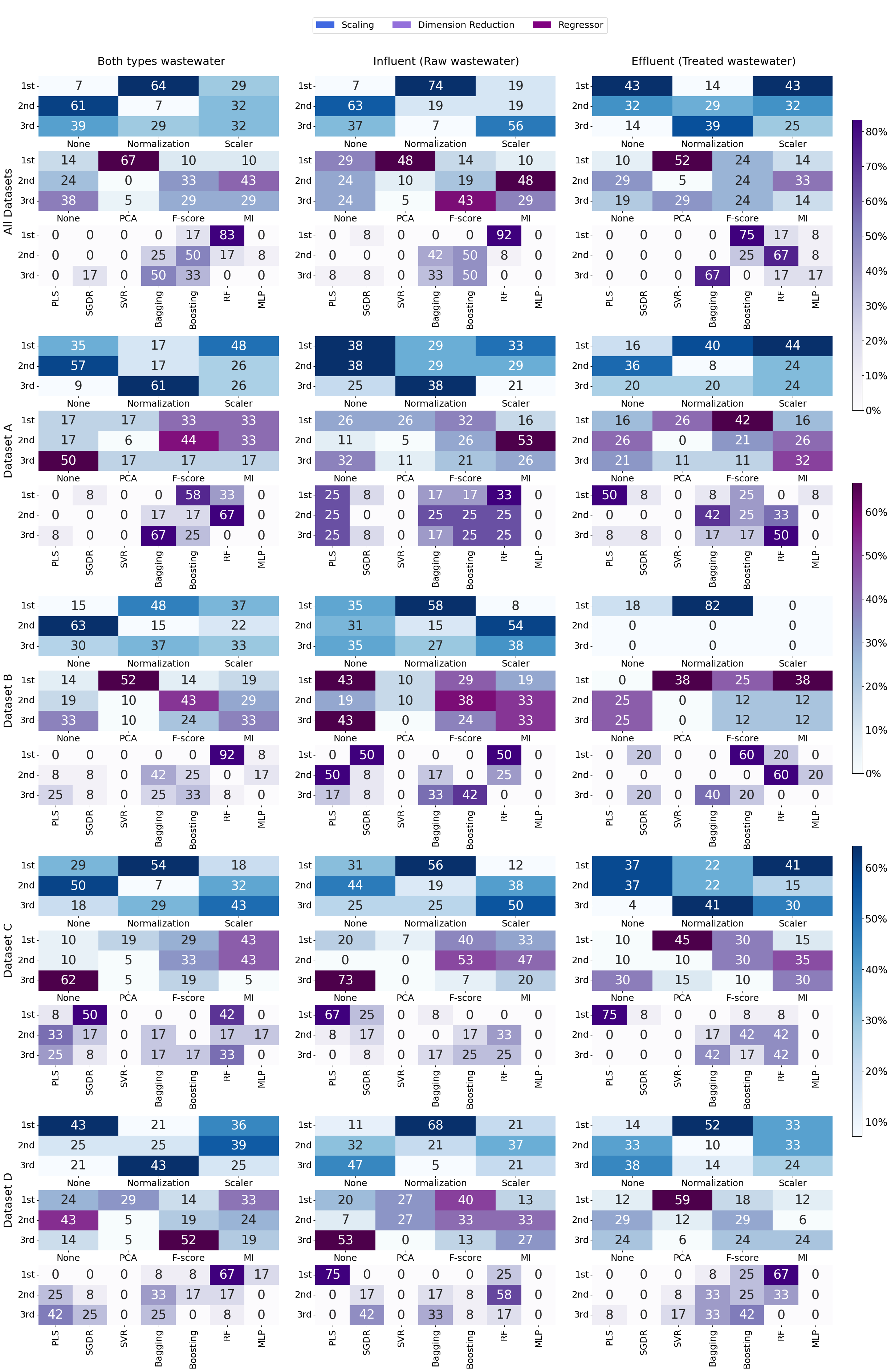** |
| --- |
| **Figure S1.** Comparative performance matrix by dataset and water type, broken down by dimension reduction, scaling, and regressor, evaluated by R2, for COD |

| **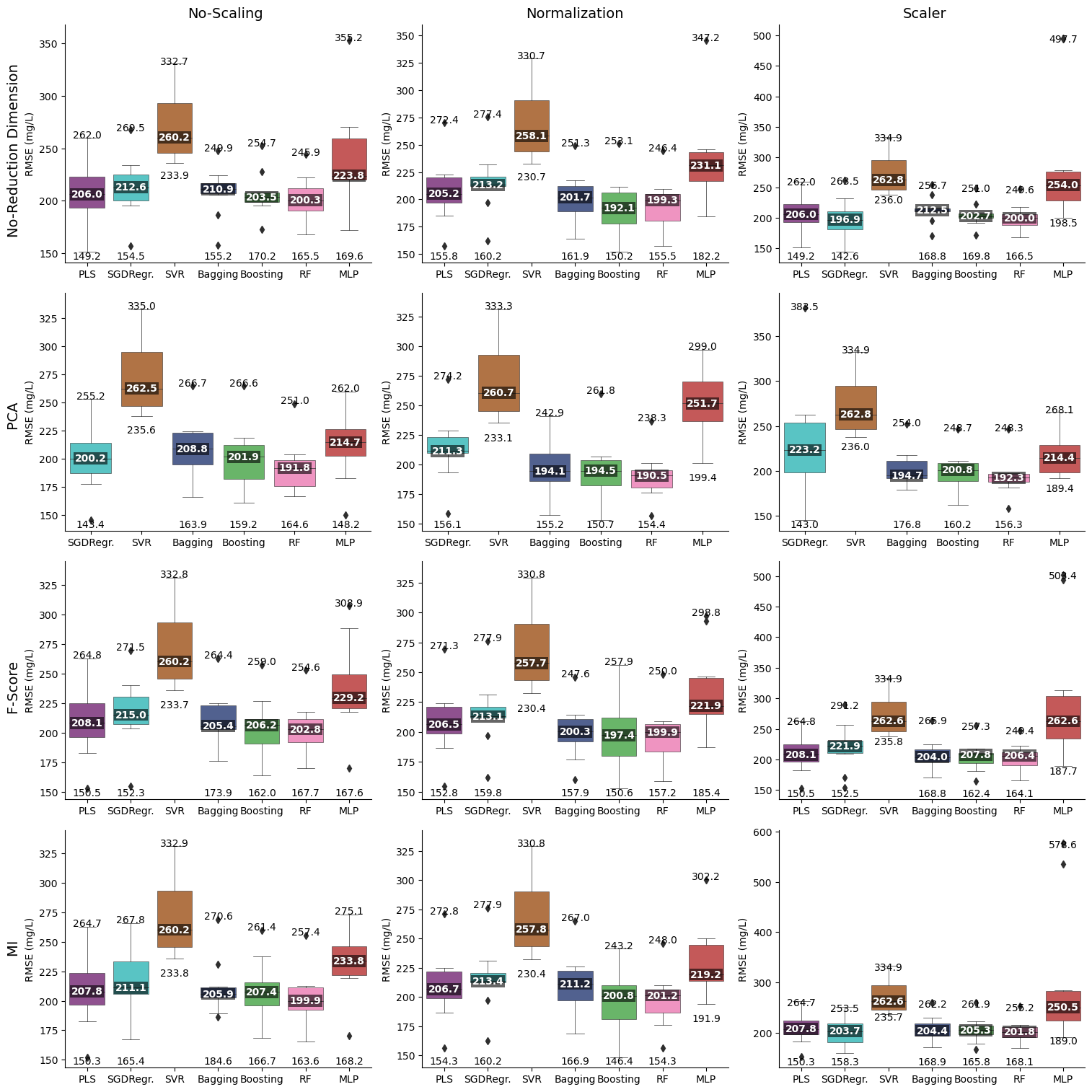** |
| --- |
| **Figure S2.** Comparative box-and-whisker plot, organized by dimension reduction and scaling, for each of the study regressors, relative to the sub-dataset L2 for COD. |

| **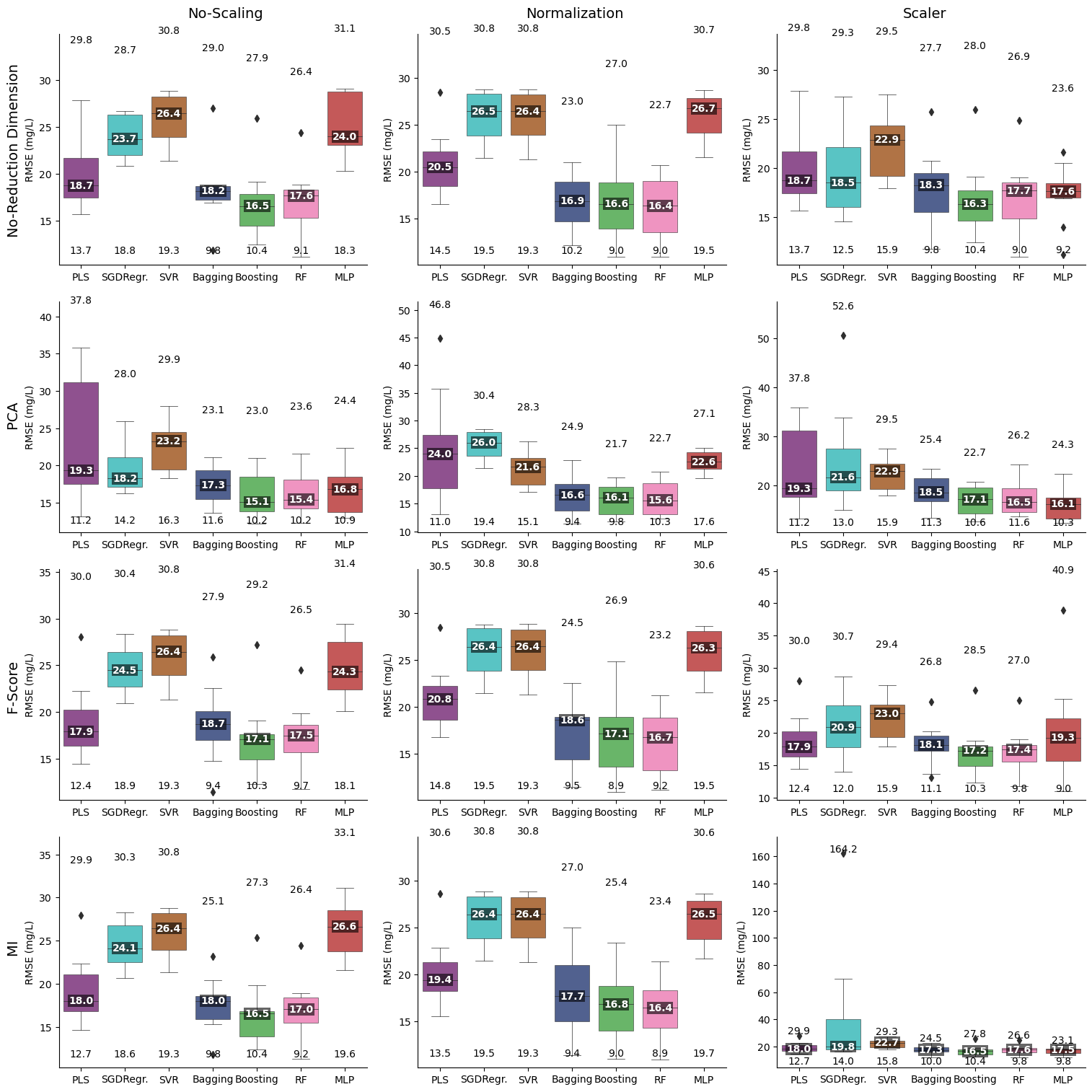** |
| --- |
| **Figure S3.** Comparative box-and-whisker plot, organized by dimension reduction and scaling, for each of the study regressors, relative to the sub-dataset L3 for COD. |

| **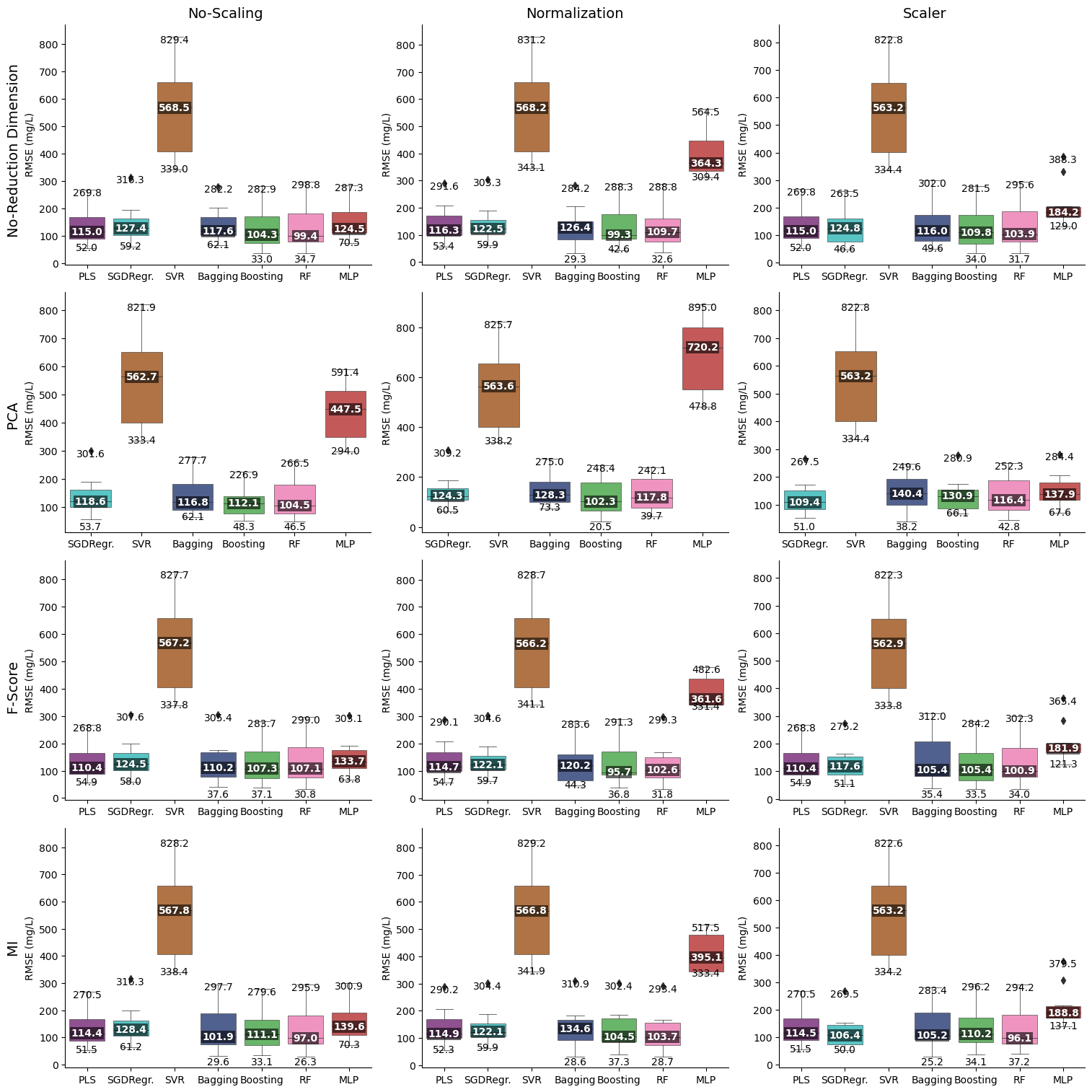** |
| --- |
| **Figure S4.** Comparative box-and-whisker plot, organized by dimension reduction and scaling, for each of the study regressors, relative to the sub-dataset A1 for COD. |

| **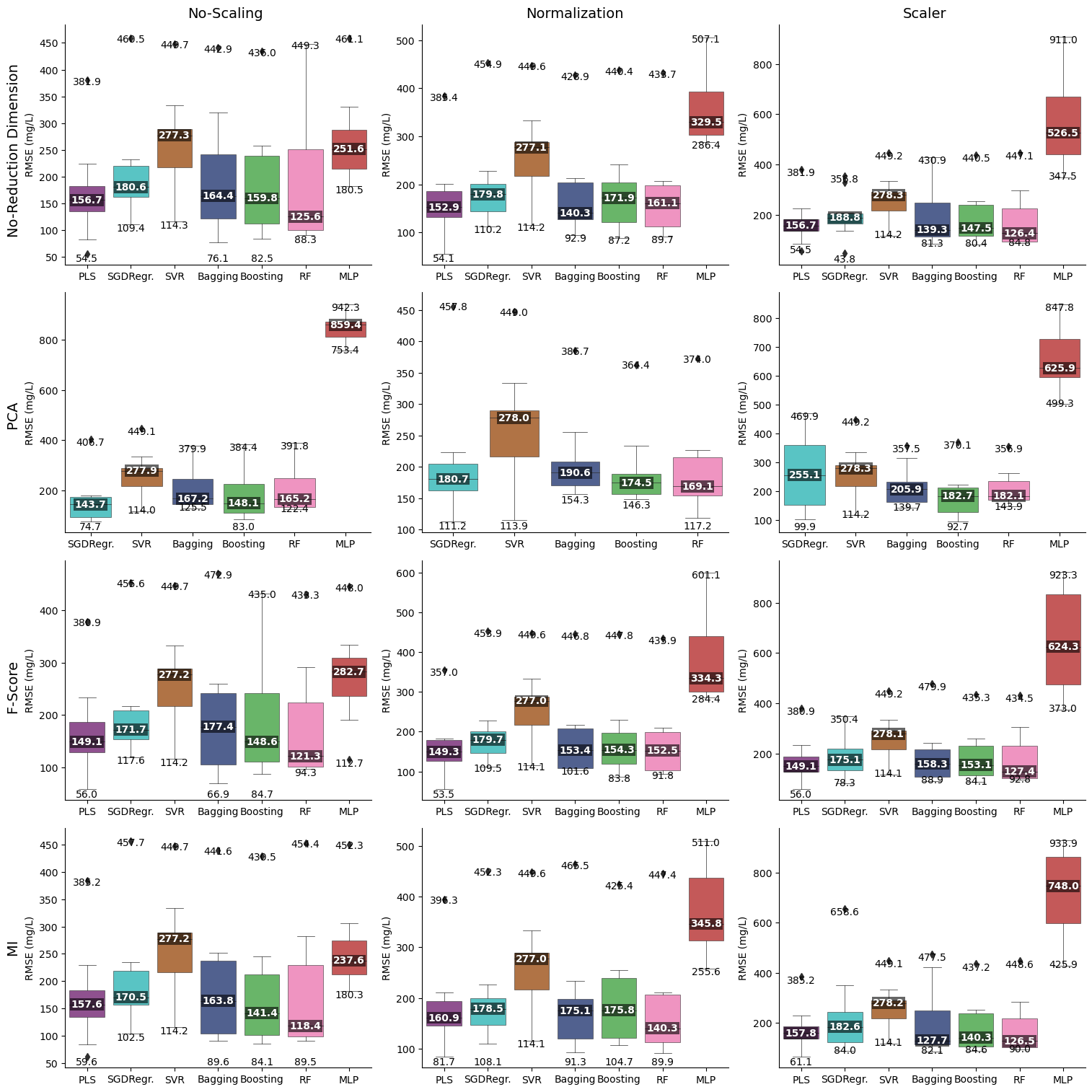** |
| --- |
| **Figure S5.** Comparative box-and-whisker plot, organized by dimension reduction and scaling, for each of the study regressors, relative to the sub-dataset A2 for COD. |

| **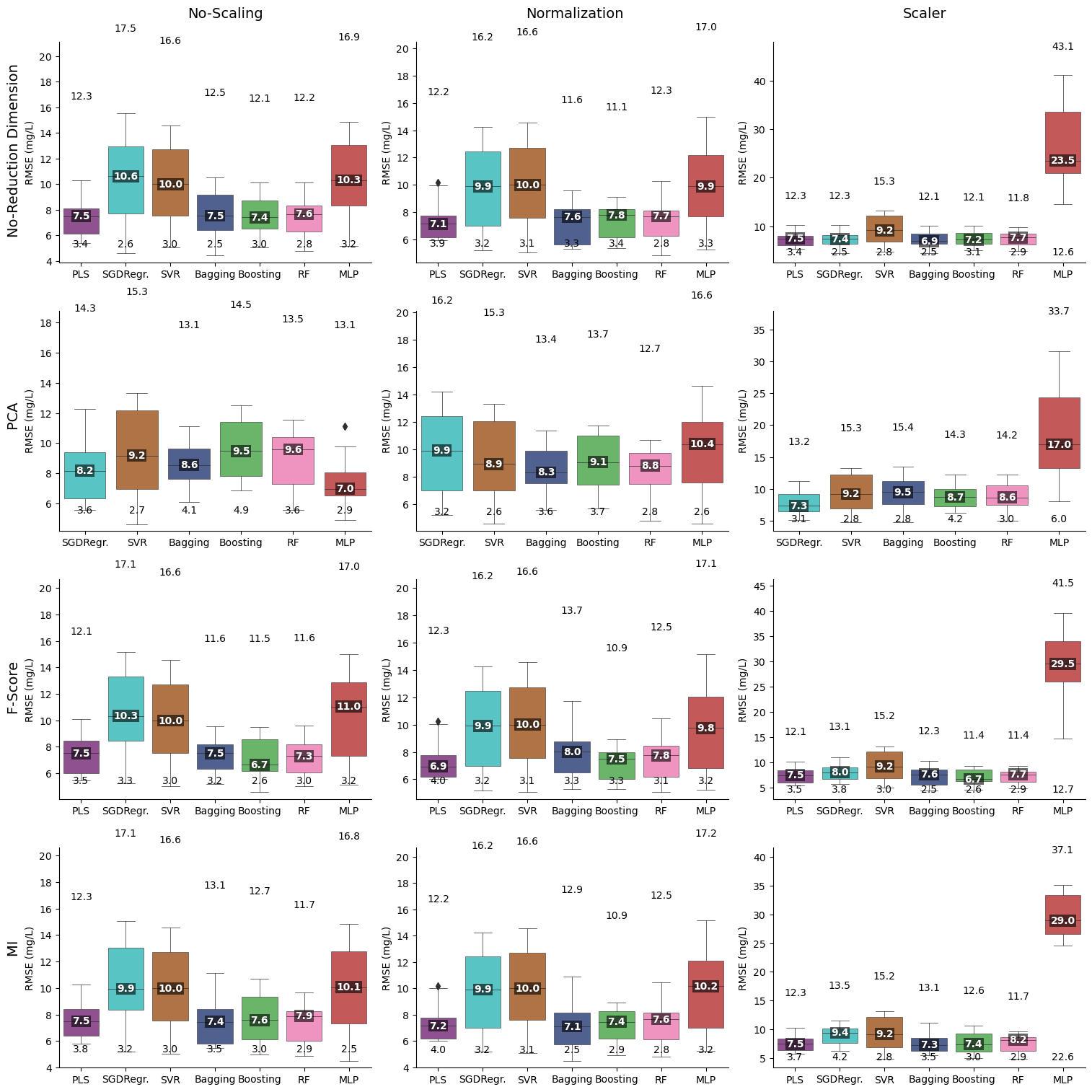** |
| --- |
| **Figure S6.** Comparative box-and-whisker plot, organized by dimension reduction and scaling, for each of the study regressors, relative to the sub-dataset A3 for COD. |

| **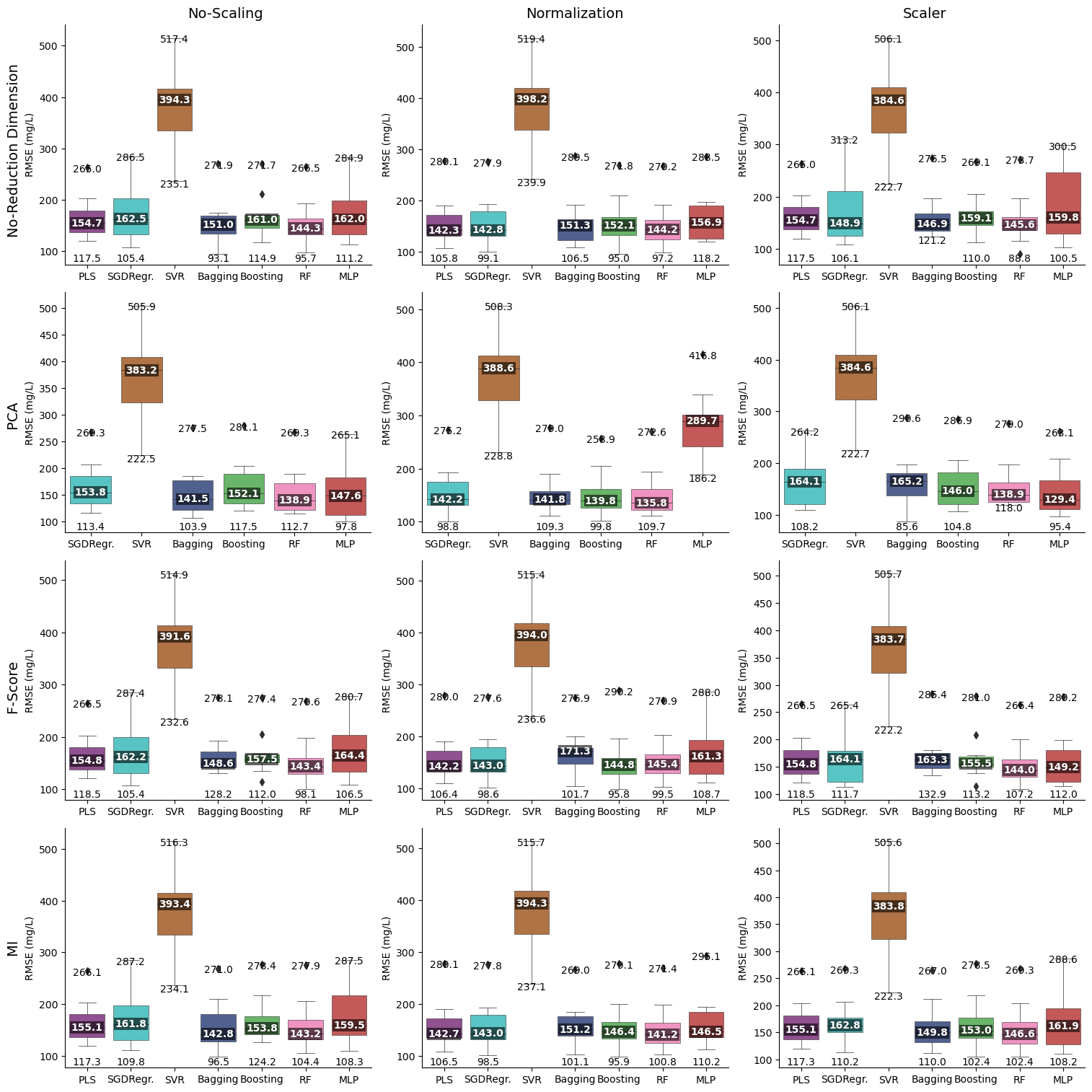** |
| --- |
| **Figure S7.** Comparative box-and-whisker plot, organized by dimension reduction and scaling, for each of the study regressors, relative to the sub-dataset B1 for COD. |

| **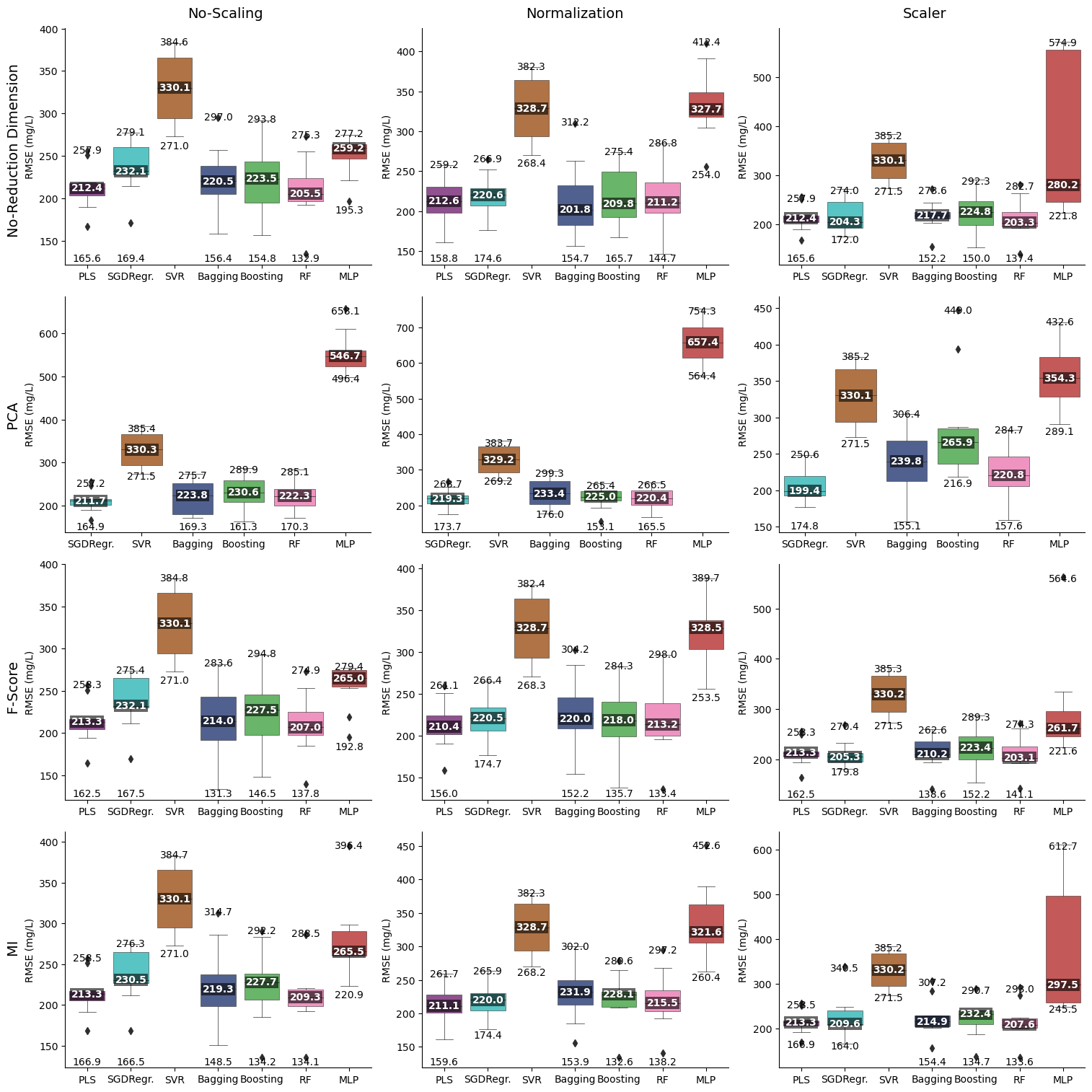** |
| --- |
| **Figure S8.** Comparative box-and-whisker plot, organized by dimension reduction and scaling, for each of the study regressors, relative to the sub-dataset B2 for COD. |

| **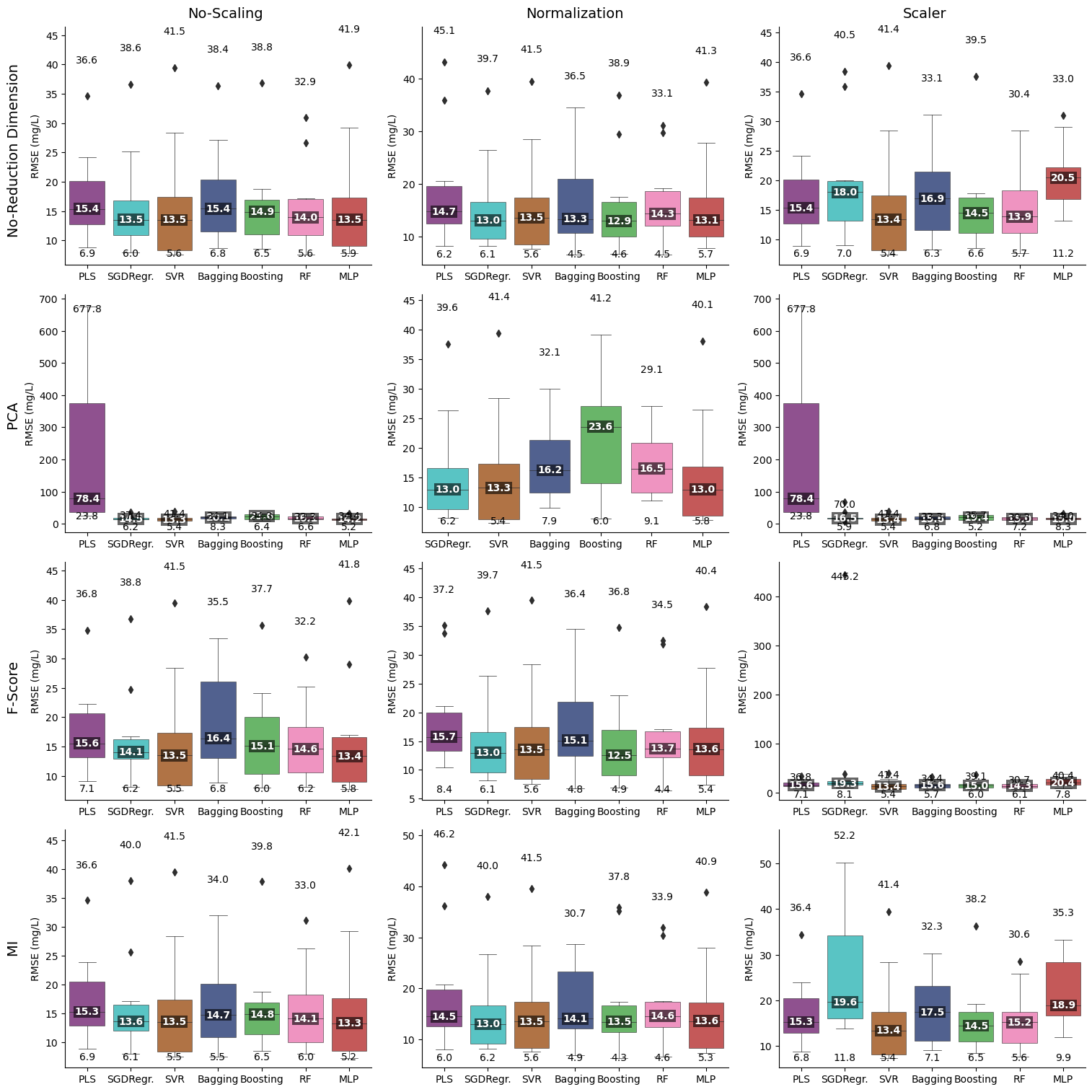** |
| --- |
| **Figure S9.** Comparative box-and-whisker plot, organized by dimension reduction and scaling, for each of the study regressors, relative to the sub-dataset B3 for COD. |

| **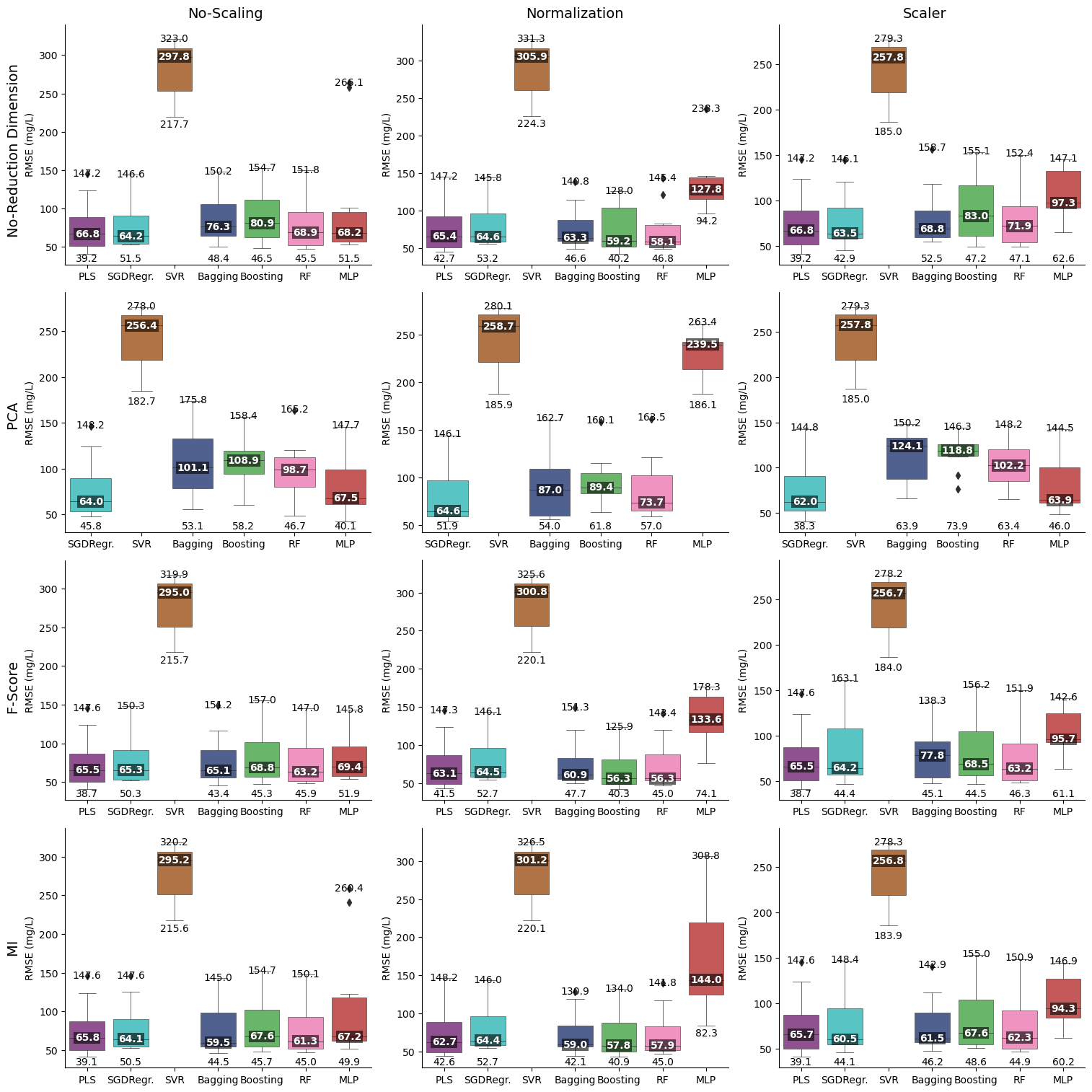** |
| --- |
| **Figure S10.** Comparative box-and-whisker plot, organized by dimension reduction and scaling, for each of the study regressors, relative to the sub-dataset C1 for COD. |

| **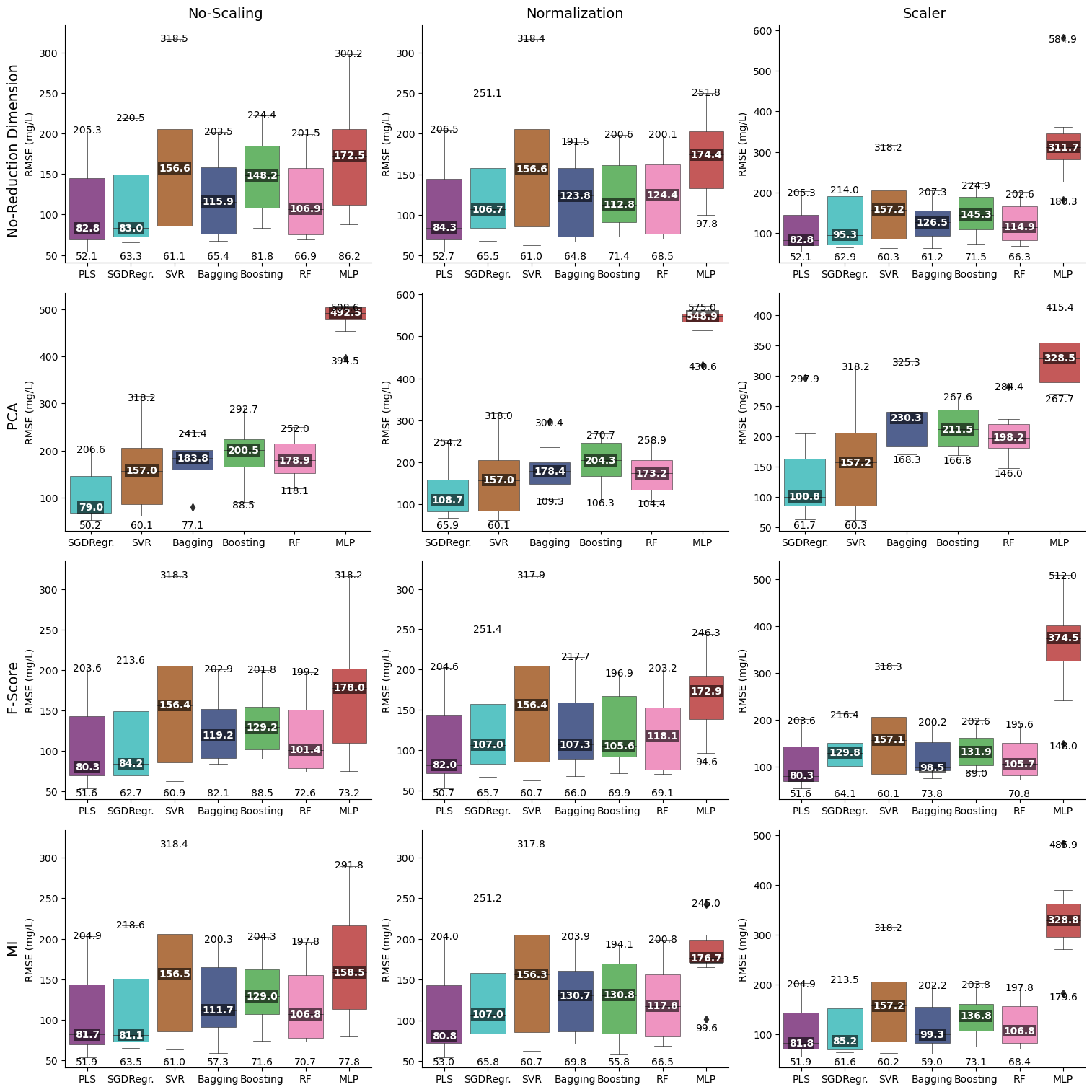** |
| --- |
| **Figure S11.** Comparative box-and-whisker plot, organized by dimension reduction and scaling, for each of the study regressors, relative to the sub-dataset C2 for COD. |

| **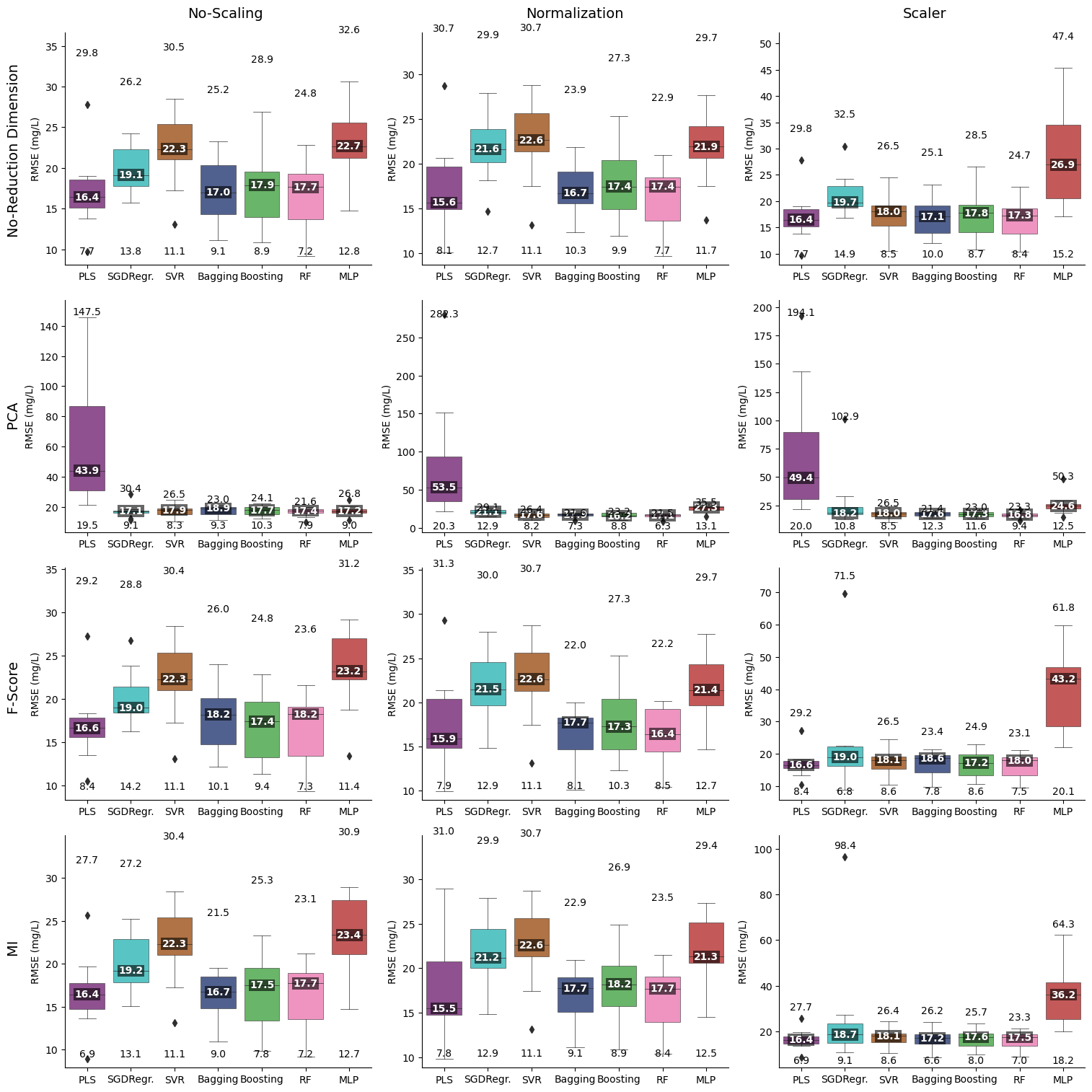** |
| --- |
| **Figure S12.** Comparative box-and-whisker plot, organized by dimension reduction and scaling, for each of the study regressors, relative to the sub-dataset C3 for COD. |

| **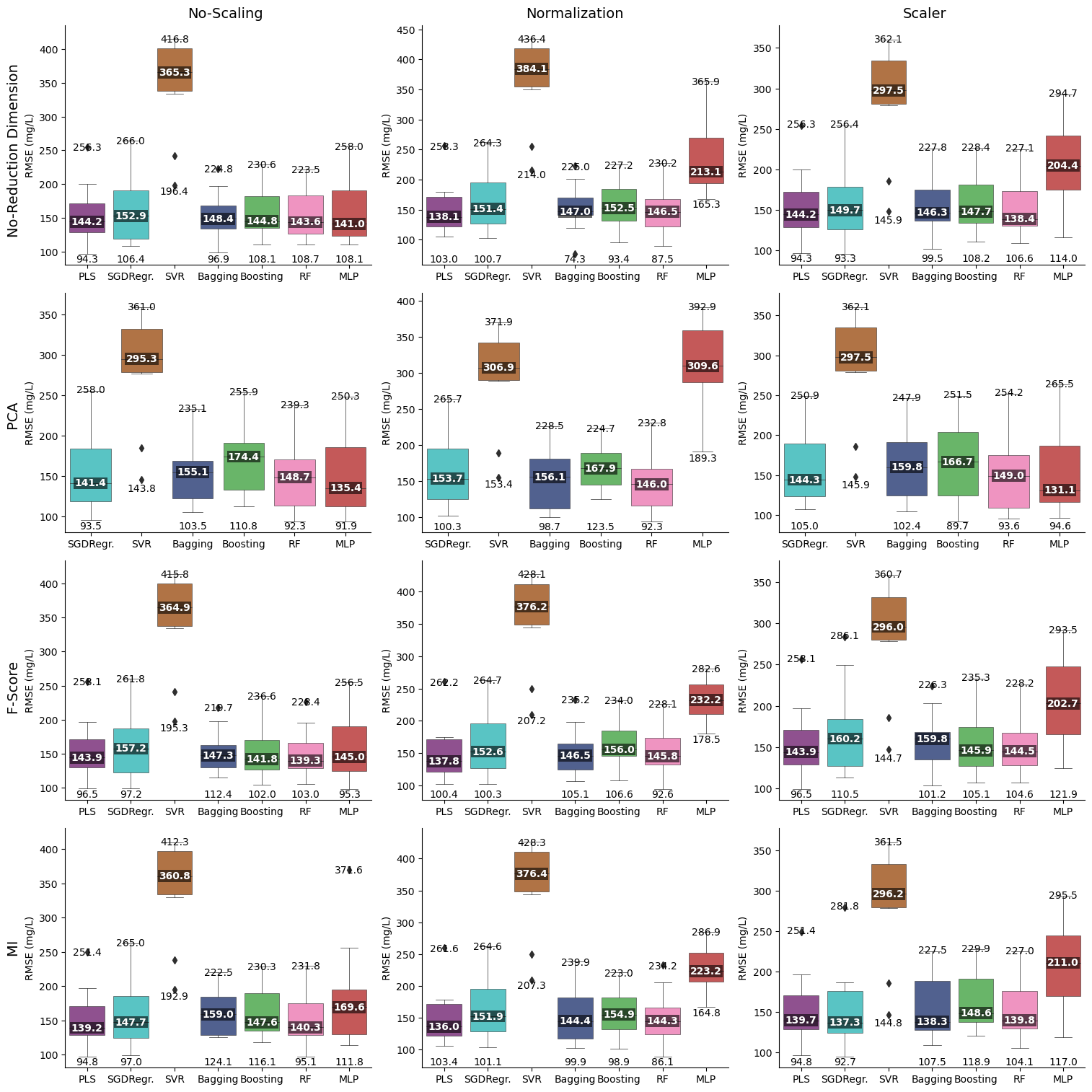** |
| --- |
| **Figure S13.** Comparative box-and-whisker plot, organized by dimension reduction and scaling, for each of the study regressors, relative to the sub-dataset D1 for COD. |

| **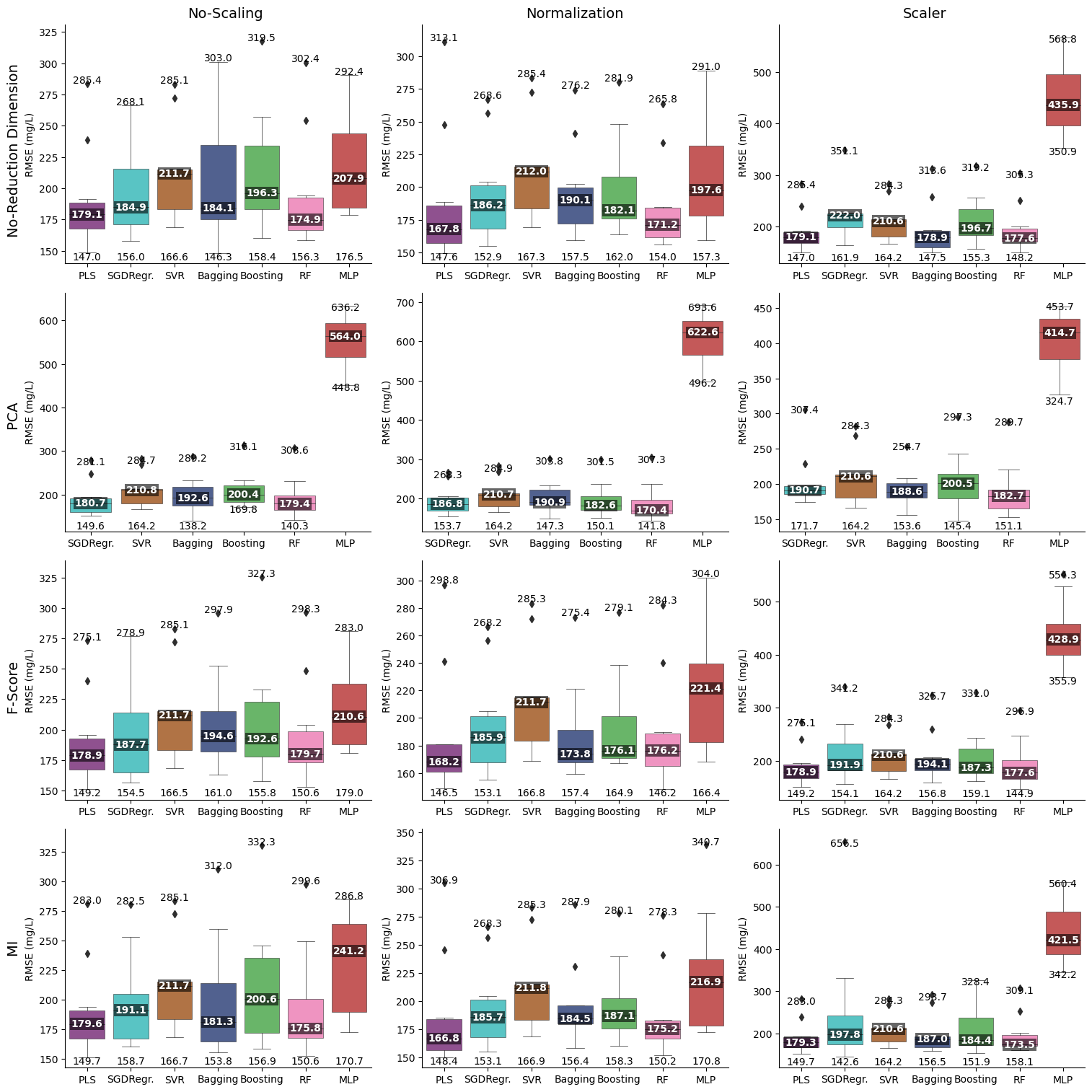** |
| --- |
| **Figure S14.** Comparative box-and-whisker plot, organized by dimension reduction and scaling, for each of the study regressors, relative to the sub-dataset D2 for COD. |

| **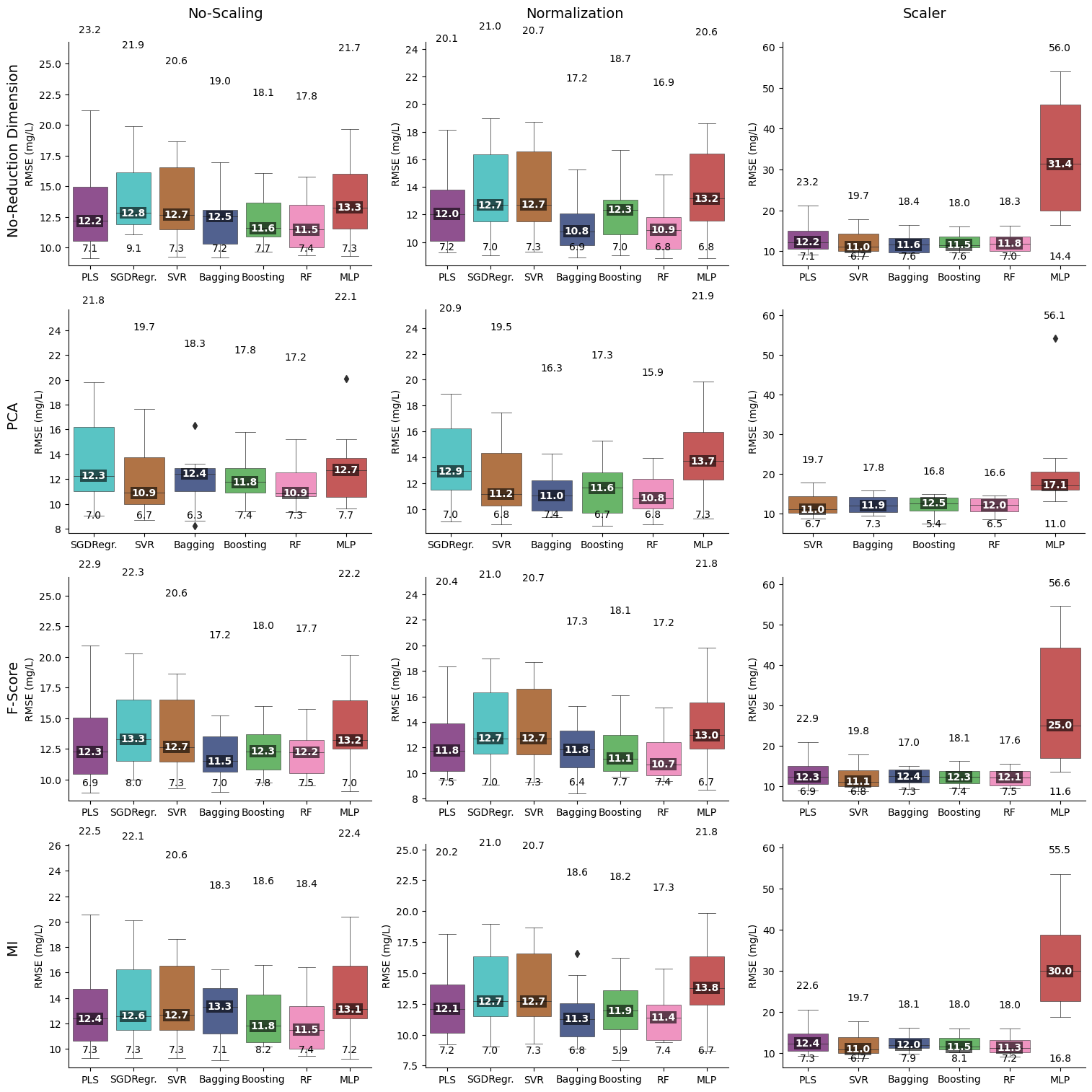** |
| --- |
| **Figure S15.** Comparative box-and-whisker plot, organized by dimension reduction and scaling, for each of the study regressors, relative to the sub-dataset D3 for COD. |

**3.2. Total Suspended Solids (TSS)**

| **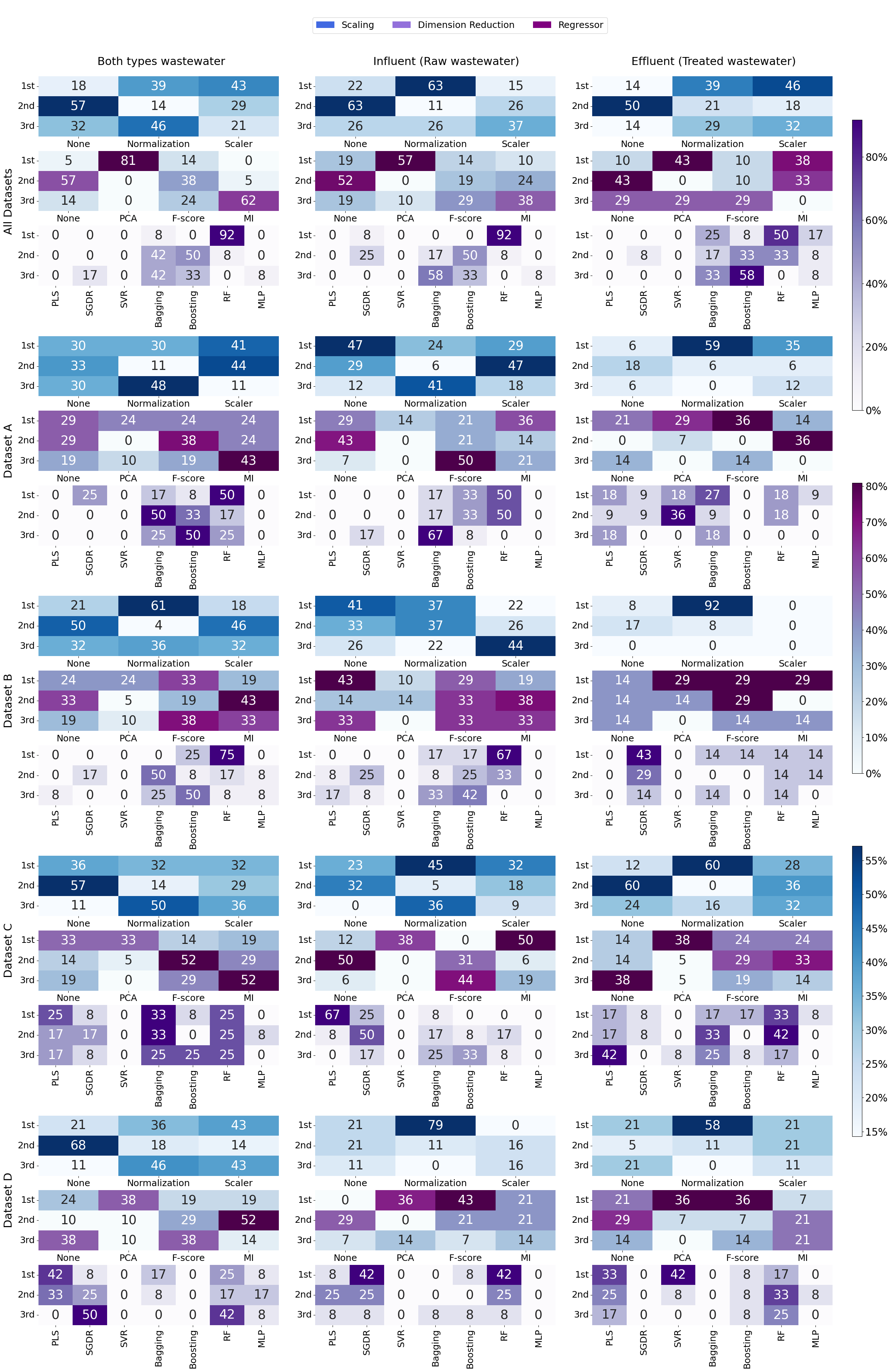** |
| --- |
| **Figure S16.** Comparative performance matrix by dataset and water type, broken down by dimension reduction, scaling, and regressor, evaluated by R2, for TSS |

| **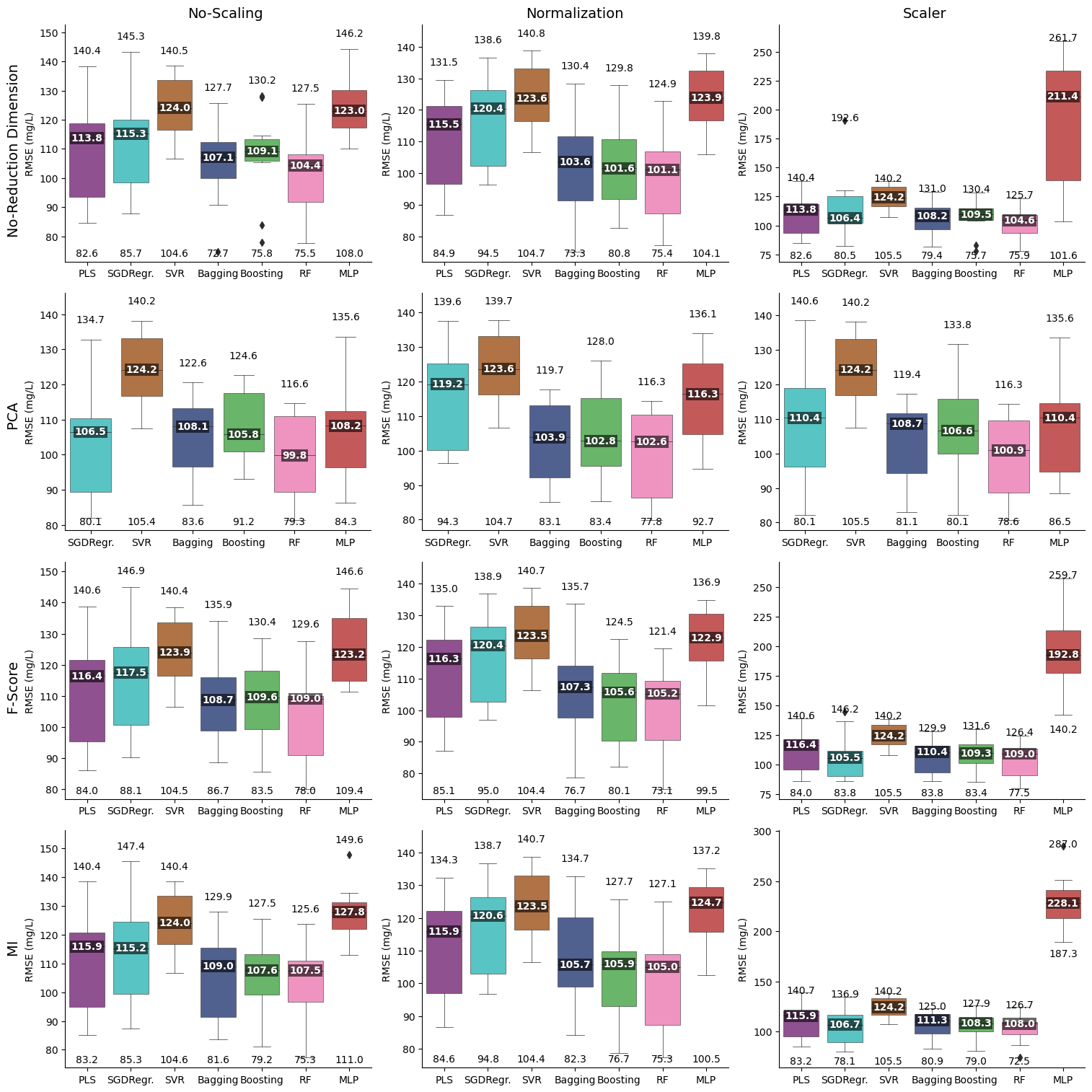** |
| --- |
| **Figure S17.** Comparative box-and-whisker plot, organized by dimension reduction and scaling, for each of the study regressors, relative to the sub-dataset L2 for TSS. |

| **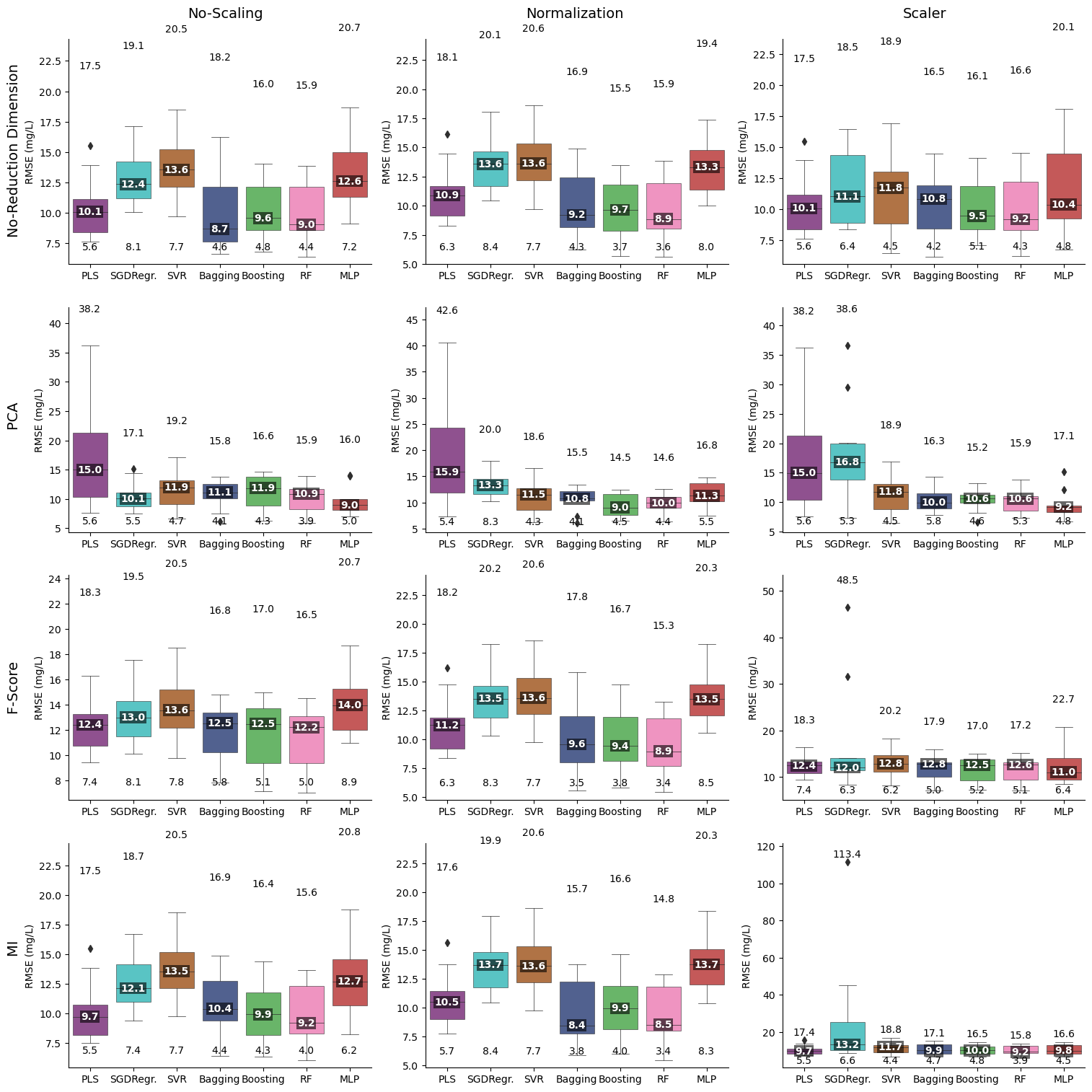** |
| --- |
| **Figure S18.** Comparative box-and-whisker plot, organized by dimension reduction and scaling, for each of the study regressors, relative to the sub-dataset L3 for TSS. |

| **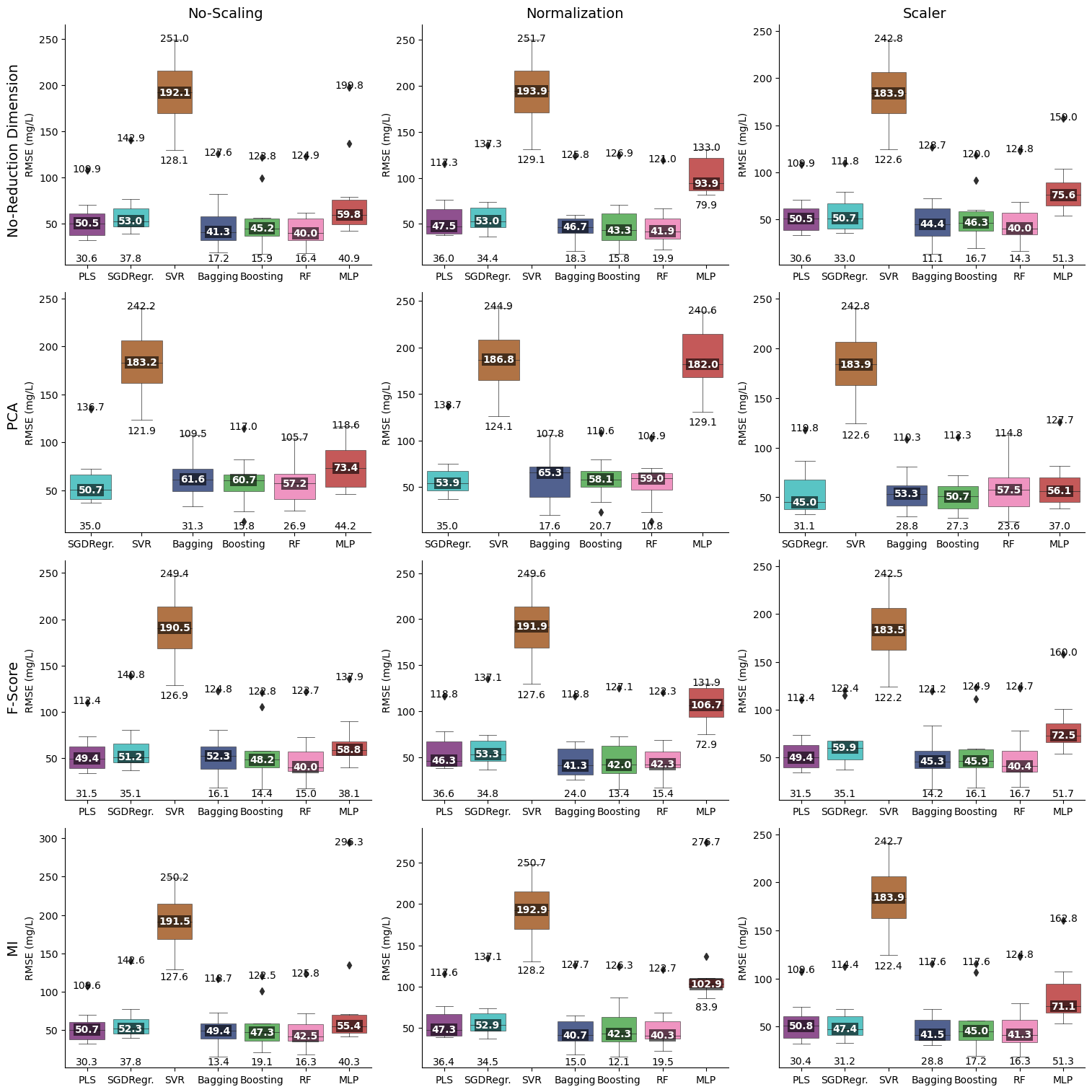** |
| --- |
| **Figure S19.** Comparative box-and-whisker plot, organized by dimension reduction and scaling, for each of the study regressors, relative to the sub-dataset A1 for TSS. |

| **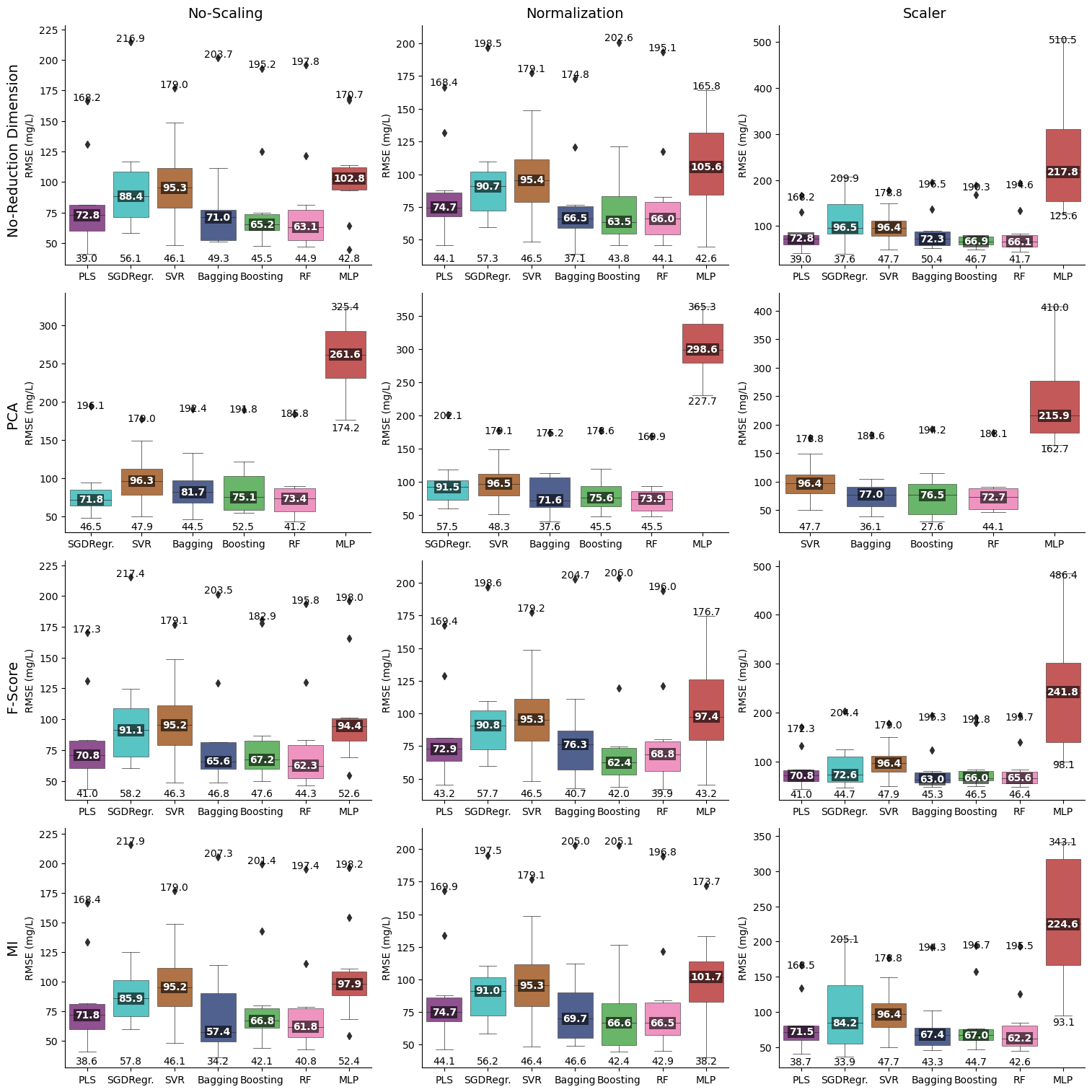** |
| --- |
| **Figure S20.** Comparative box-and-whisker plot, organized by dimension reduction and scaling, for each of the study regressors, relative to the sub-dataset A2 for TSS. |

| **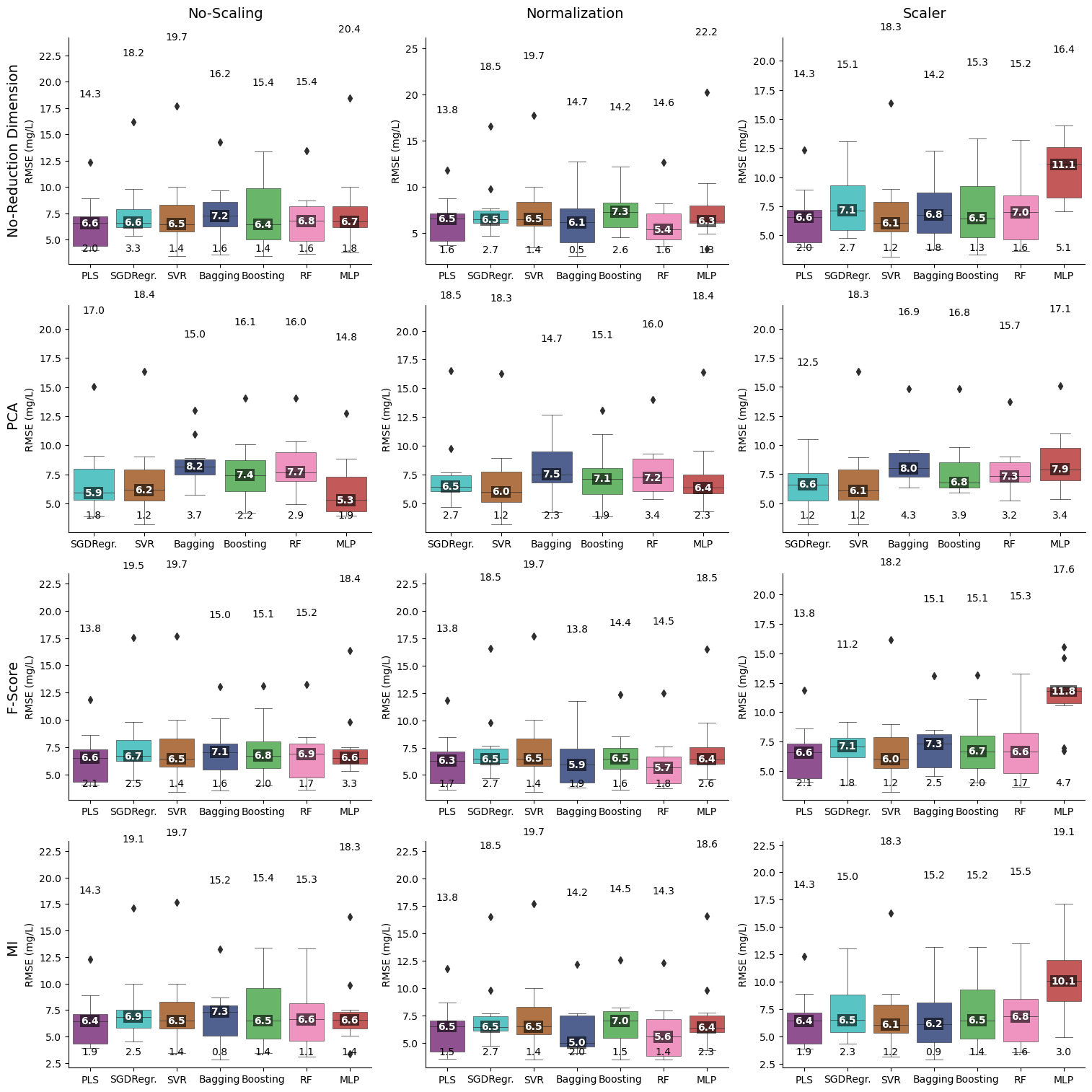** |
| --- |
| **Figure S21.** Comparative box-and-whisker plot, organized by dimension reduction and scaling, for each of the study regressors, relative to the sub-dataset A3 for TSS. |

| **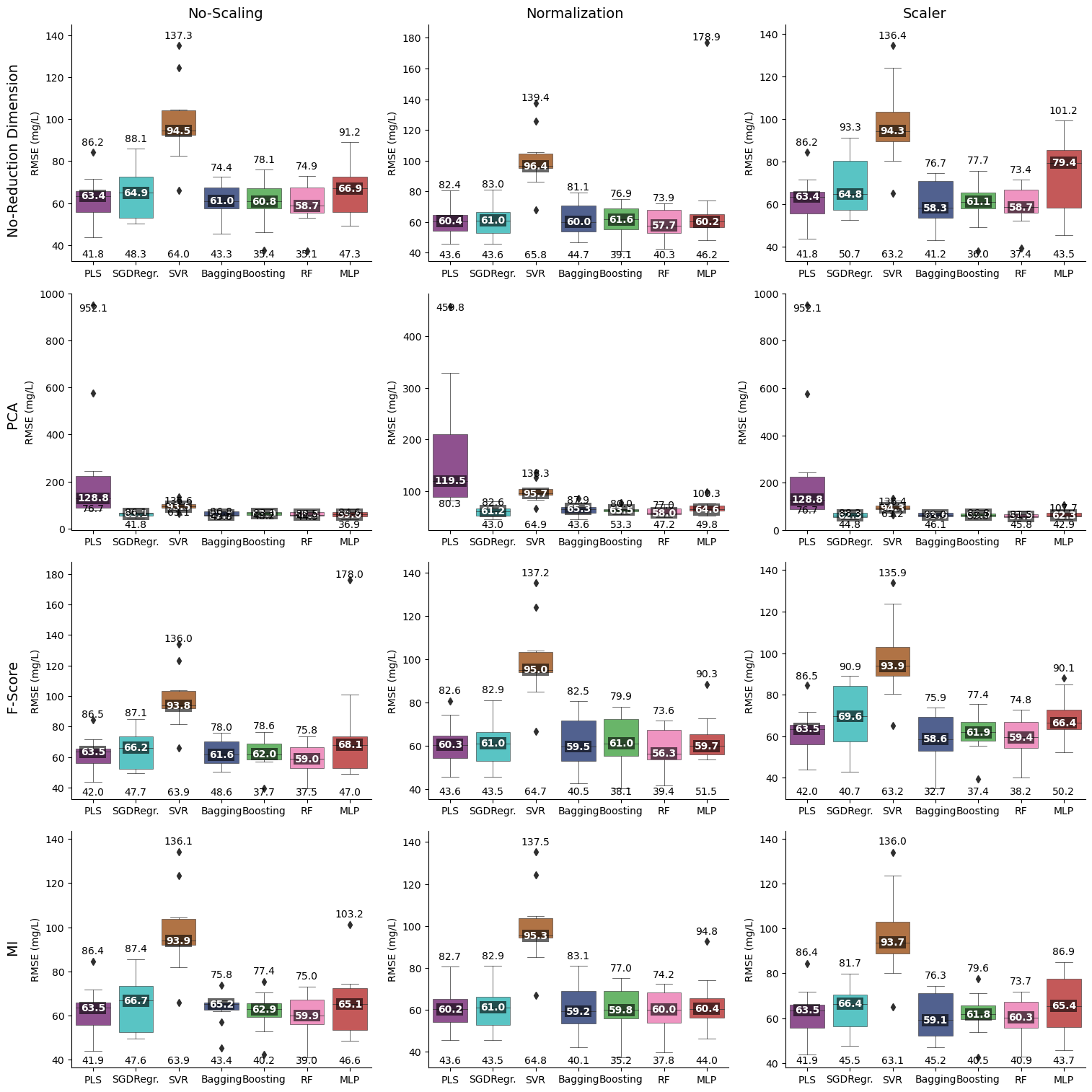** |
| --- |
| **Figure S22.** Comparative box-and-whisker plot, organized by dimension reduction and scaling, for each of the study regressors, relative to the sub-dataset B1 for TSS. |

| **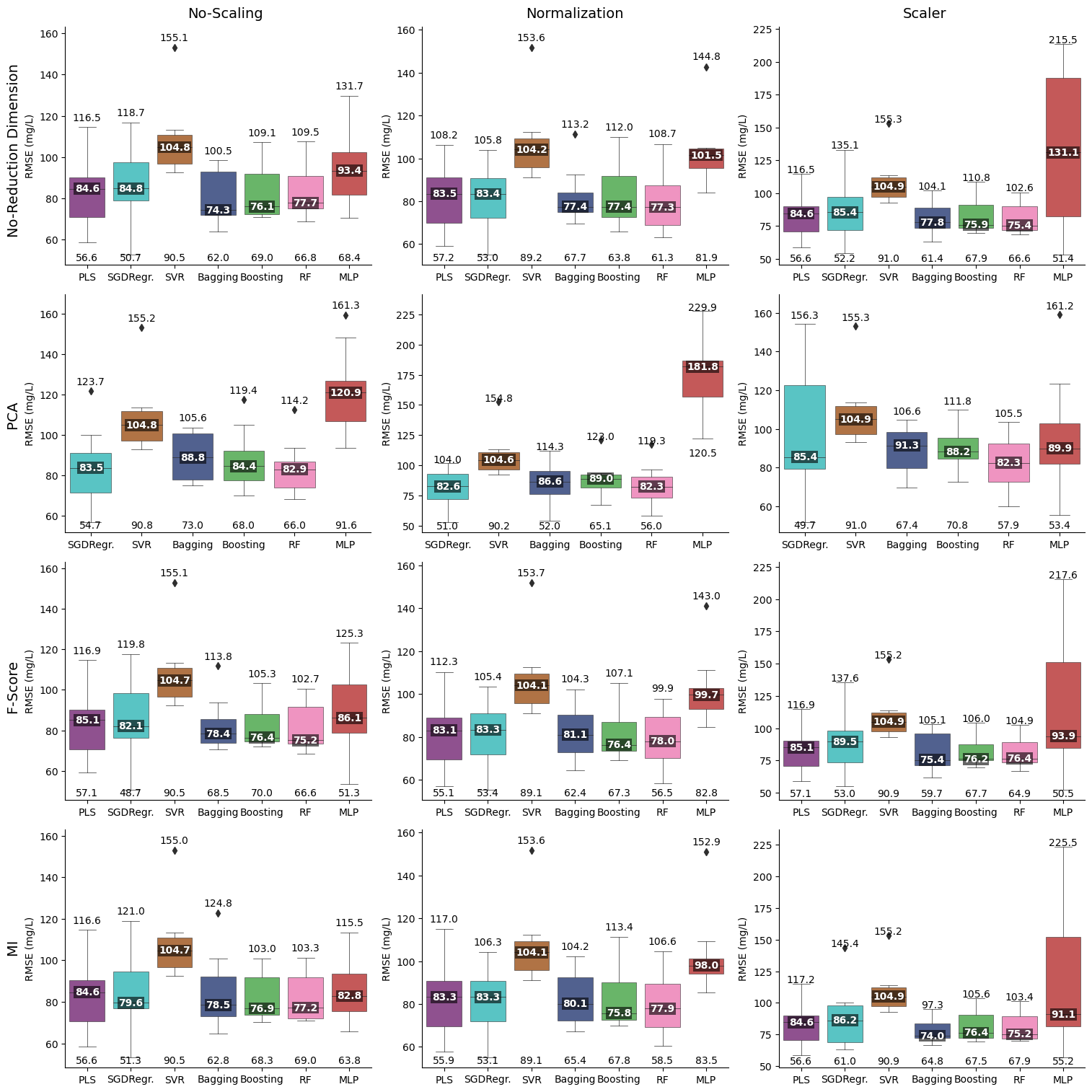** |
| --- |
| **Figure S23.** Comparative box-and-whisker plot, organized by dimension reduction and scaling, for each of the study regressors, relative to the sub-dataset B2 for TSS. |

| **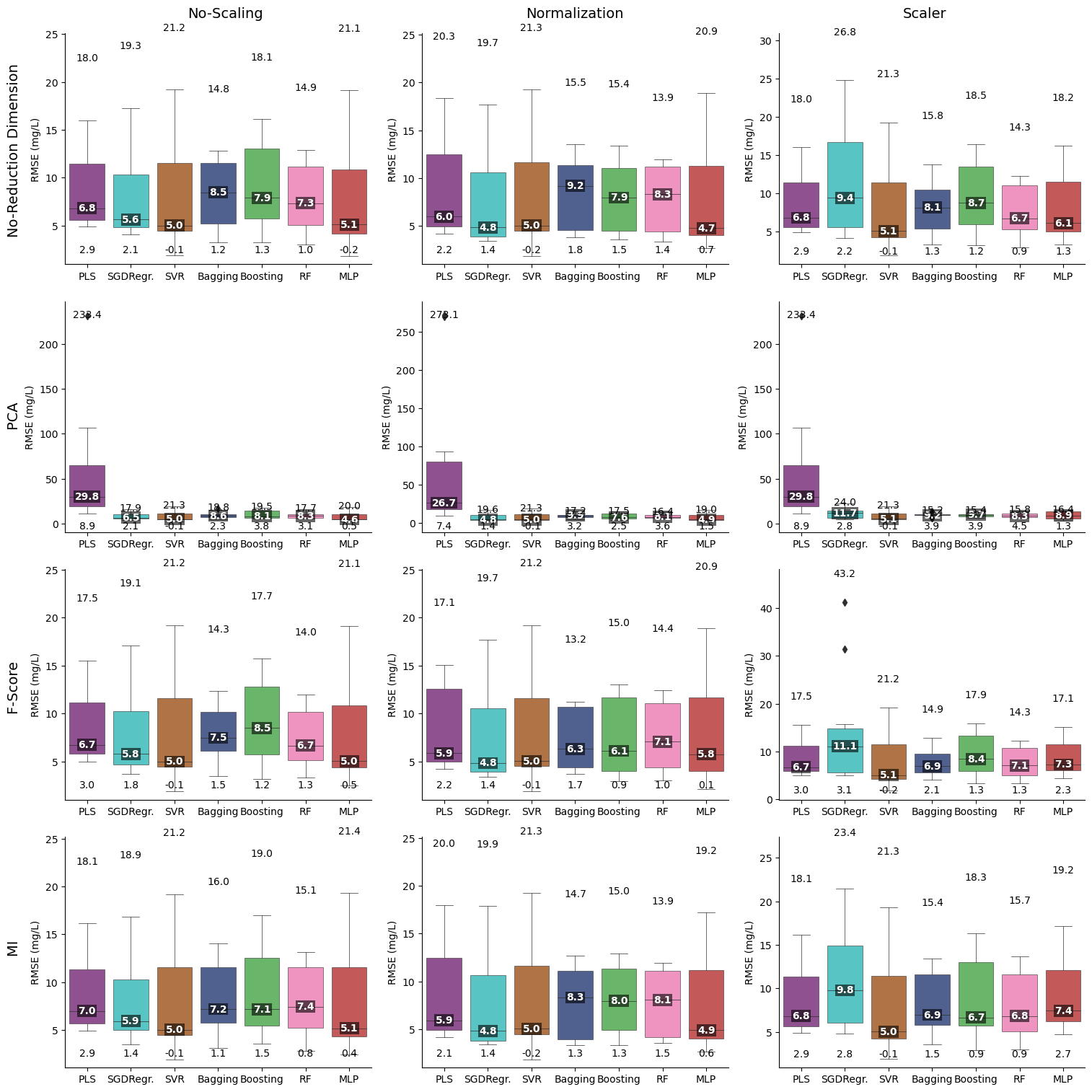** |
| --- |
| **Figure S24.** Comparative box-and-whisker plot, organized by dimension reduction and scaling, for each of the study regressors, relative to the sub-dataset B3 for TSS. |

| **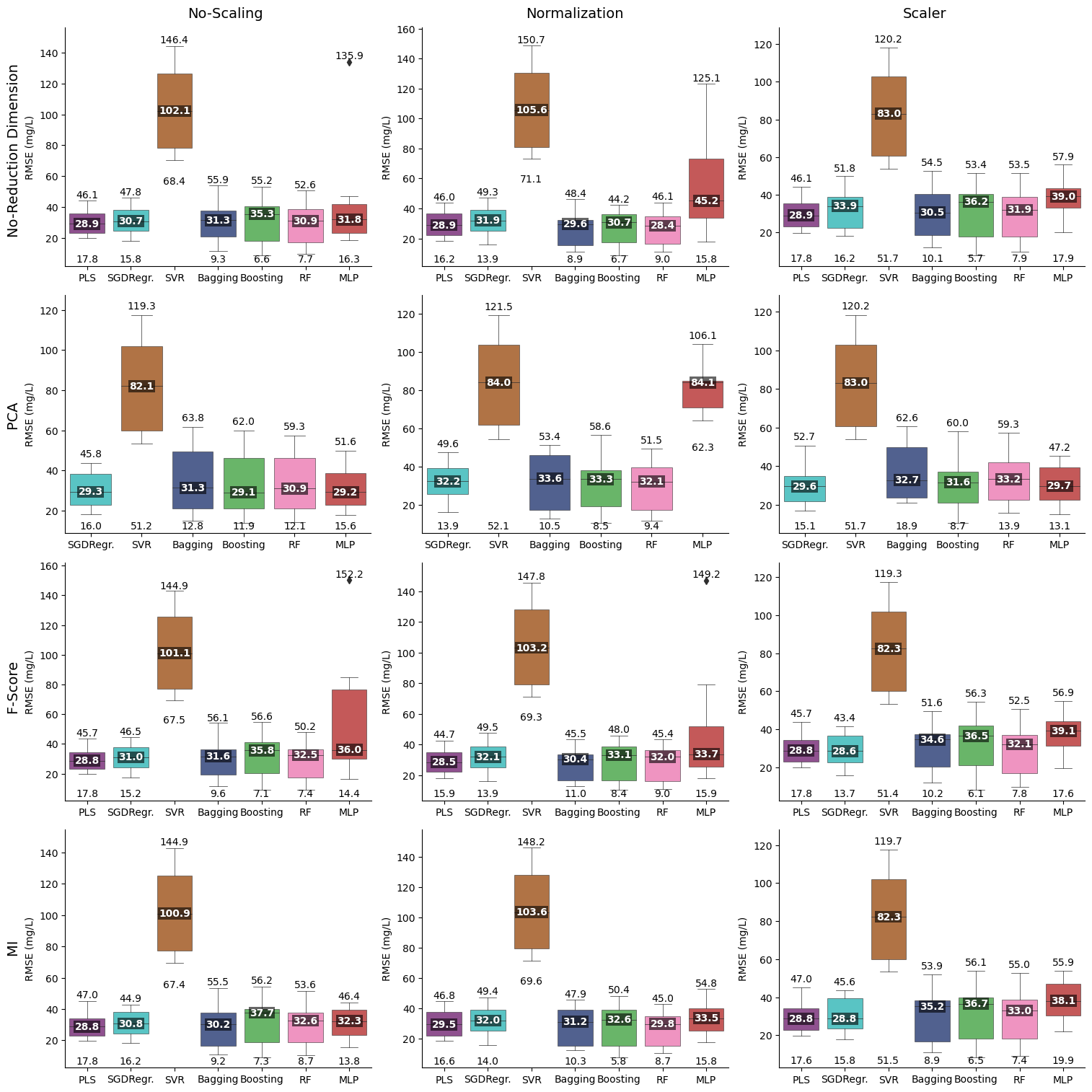** |
| --- |
| **Figure S25.** Comparative box-and-whisker plot, organized by dimension reduction and scaling, for each of the study regressors, relative to the sub-dataset C1 for TSS. |

| **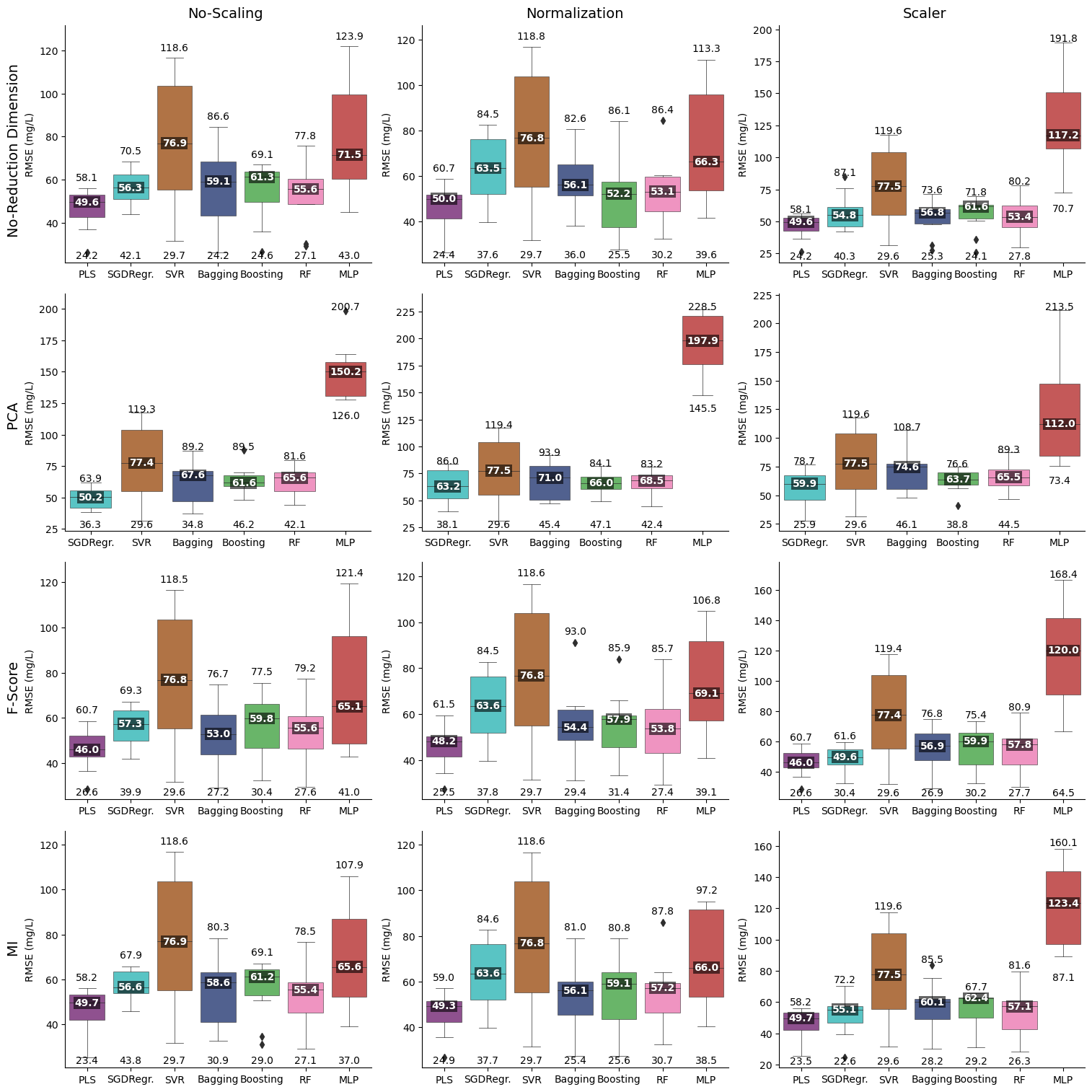** |
| --- |
| **Figure S26.** Comparative box-and-whisker plot, organized by dimension reduction and scaling, for each of the study regressors, relative to the sub-dataset C2 for TSS. |

| **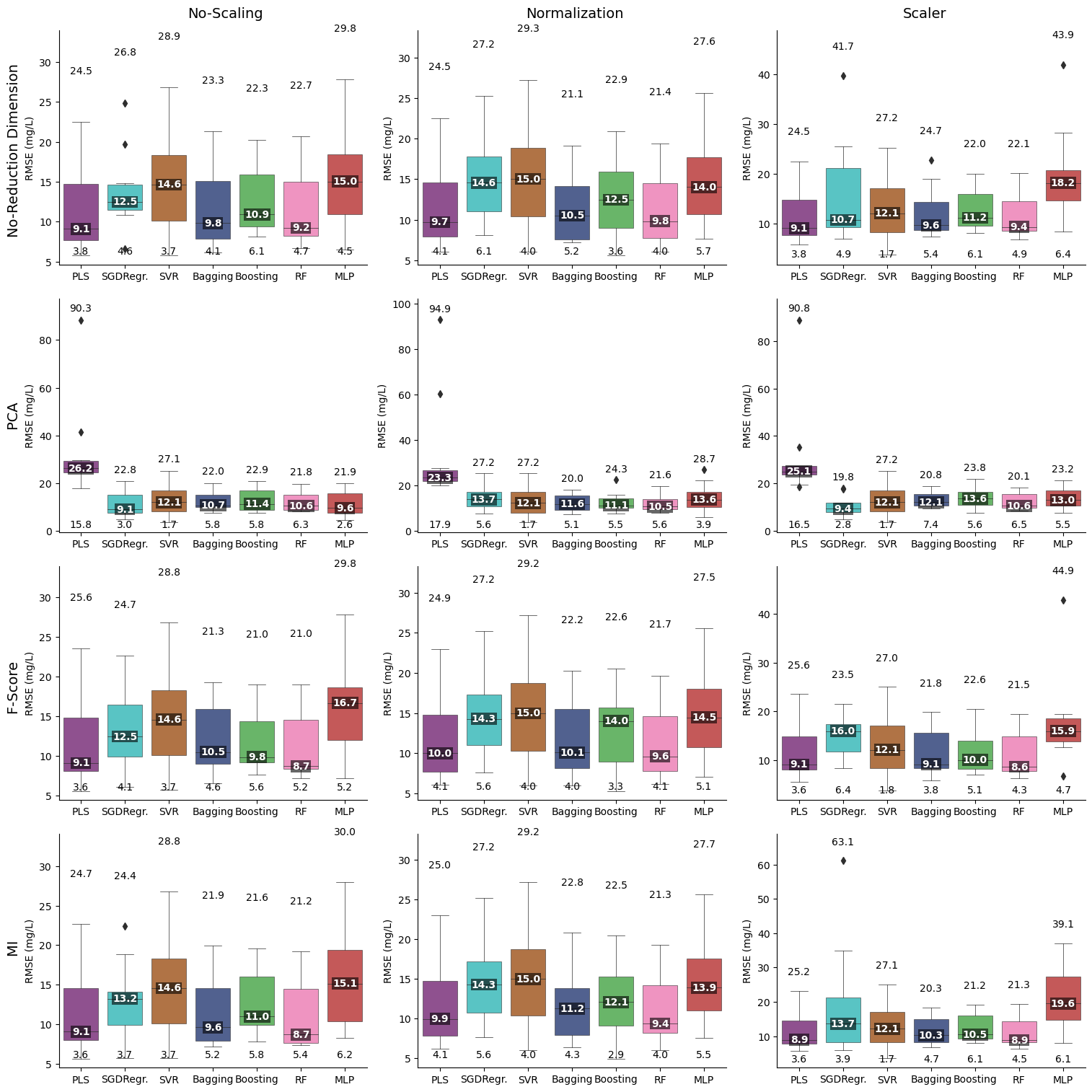** |
| --- |
| **Figure S27.** Comparative box-and-whisker plot, organized by dimension reduction and scaling, for each of the study regressors, relative to the sub-dataset C3 for TSS. |

| **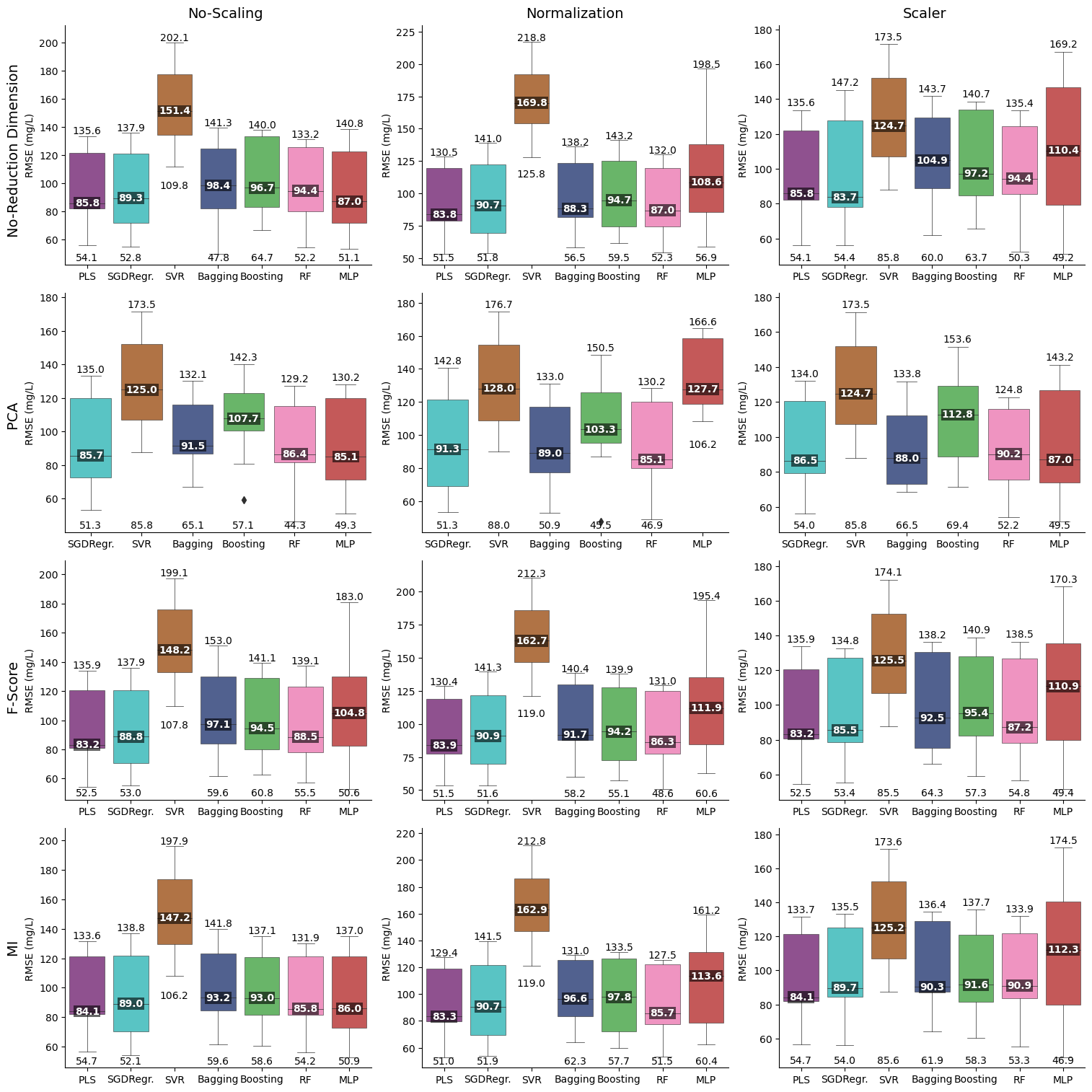** |
| --- |
| **Figure S28.** Comparative box-and-whisker plot, organized by dimension reduction and scaling, for each of the study regressors, relative to the sub-dataset D1 for TSS. |

| **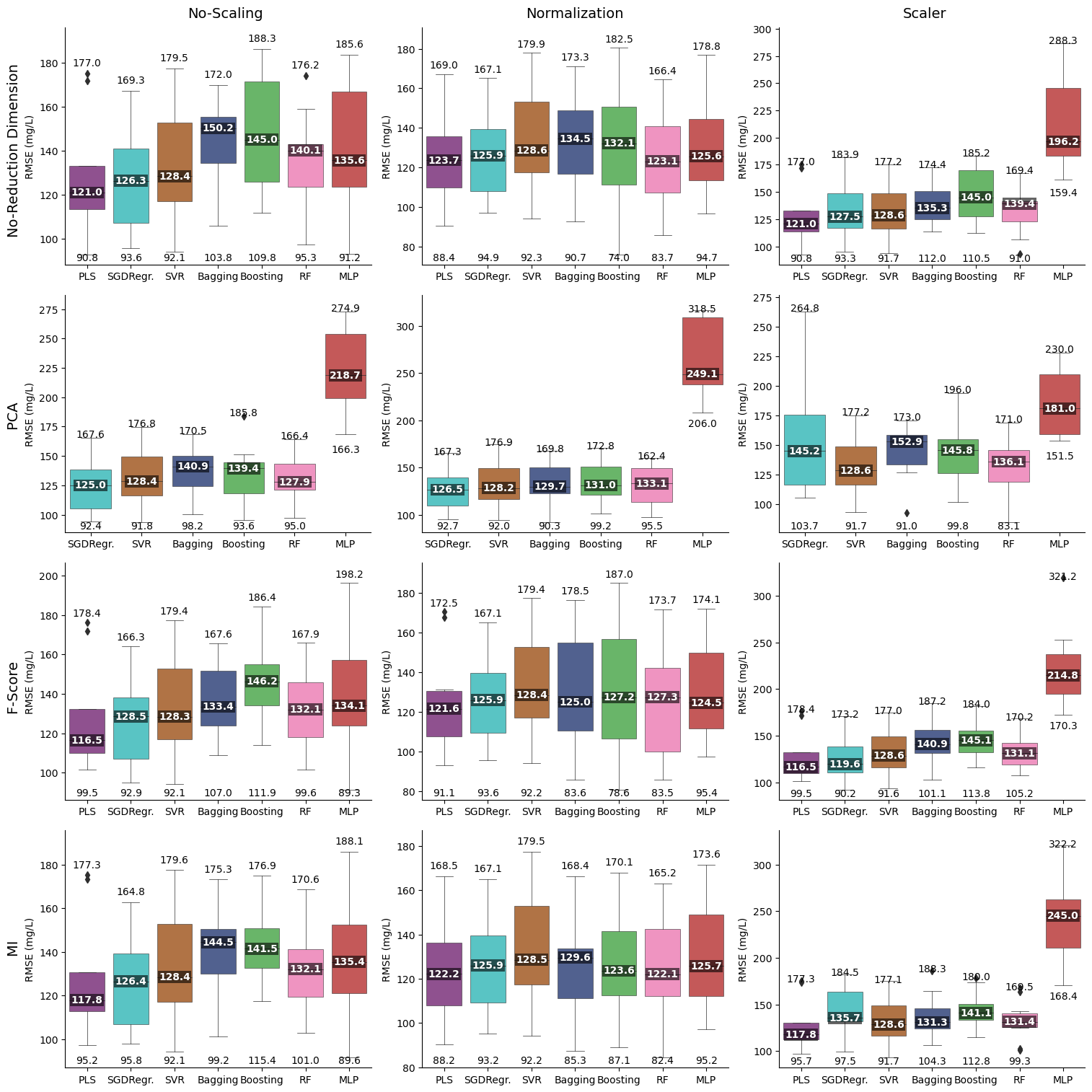** |
| --- |
| **Figure S29.** Comparative box-and-whisker plot, organized by dimension reduction and scaling, for each of the study regressors, relative to the sub-dataset D2 for TSS. |

| **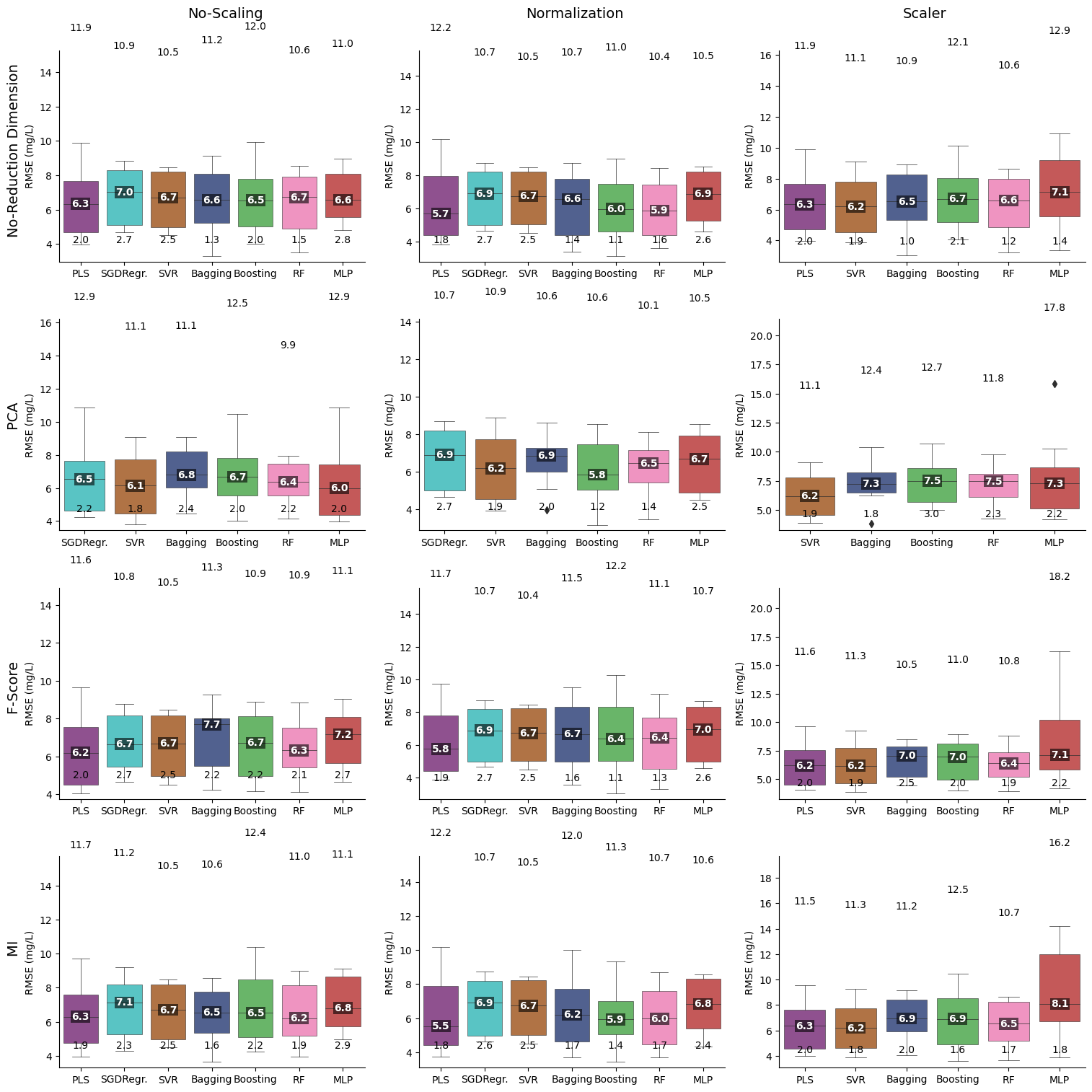** |
| --- |
| **Figure S30.** Comparative box-and-whisker plot, organized by dimension reduction and scaling, for each of the study regressors, relative to the sub-dataset D3 for TSS. |
